# Automatic identification of variables in epidemiological datasets using logic regression

**DOI:** 10.1186/s12911-017-0429-1

**Published:** 2017-04-13

**Authors:** Matthias W. Lorenz, Negin Ashtiani Abdi, Frank Scheckenbach, Anja Pflug, Alpaslan Bülbül, Alberico L. Catapano, Stefan Agewall, Marat Ezhov, Michiel L. Bots, Stefan Kiechl, Andreas Orth, on behalf of the PROG-IMT study group, Giuseppe D. Norata, Jean Philippe Empana, Hung-Ju Lin, Stela McLachlan, Lena Bokemark, Kimmo Ronkainen, Mauro Amato, Ulf Schminke, Sathanur R. Srinivasan, Lars Lind, Akihiko Kato, Chrystosomos Dimitriadis, Tadeusz Przewlocki, Shuhei Okazaki, C. D. A. Stehouwer, Tatjana Lazarevic, Peter Willeit, David N. Yanez, Helmuth Steinmetz, Dirk Sander, Holger Poppert, Moise Desvarieux, M. Arfan Ikram, Sebastjan Bevc, Daniel Staub, Cesare R. Sirtori, Bernhard Iglseder, Gunnar Engström, Giovanni Tripepi, Oscar Beloqui, Moo-Sik Lee, Alfonsa Friera, Wuxiang Xie, Liliana Grigore, Matthieu Plichart, Ta-Chen Su, Christine Robertson, Caroline Schmidt, Tomi-Pekka Tuomainen, Fabrizio Veglia, Henry Völzke, Giel Nijpels, Aleksandar Jovanovic, Johann Willeit, Ralph L. Sacco, Oscar H. Franco, Radovan Hojs, Heiko Uthoff, Bo Hedblad, Hyun Woong Park, Carmen Suarez, Dong Zhao, Alberico Catapano, Pierre Ducimetiere, Kuo-Liong Chien, Jackie F. Price, Göran Bergström, Jussi Kauhanen, Elena Tremoli, Marcus Dörr, Gerald Berenson, Aikaterini Papagianni, Anna Kablak-Ziembicka, Kazuo Kitagawa, Jaqueline M. Dekker, Radojica Stolic, Stefan Kiechl, Joseph F. Polak, Matthias Sitzer, Horst Bickel, Tatjana Rundek, Albert Hofman, Robert Ekart, Beat Frauchiger, Samuela Castelnuovo, Maria Rosvall, Carmine Zoccali, Manuel F. Landecho, Jang-Ho Bae, Rafael Gabriel, Jing Liu, Damiano Baldassarre, Maryam Kavousi

**Affiliations:** 1 Department of Neurology, University Clinic Frankfurt, Schleusenweg 2-16, D-60528 Frankfurt/Main, Germany; 2 Faculty of Computer Science and Engineering, Frankfurt University of Applied Sciences, Frankfurt/Main, Germany; 3 IRCSS Multimedica, Milan, Italy; 4 Department of Pharmacological and Biomolecular Sciences, University of Milan, Milan, Italy; 5 Institute of Clinical Sciences, University of Oslo, Oslo, Norway; 6 Department of Cardiology, Oslo University Hospital Ullevål, Oslo, Norway; 7 Atherosclerosis Department, Cardiology Research Center, Moscow, Russia; 8 University Medical Center Utrecht, Utrecht, The Netherlands; 9 Department of Epidemiology and Biostatistics, Erasmus Medical Center, Rotterdam, The Netherlands; 10 Department of Neurology, Medical University Innsbruck, Innsbruck, Austria

**Keywords:** Meta-analysis, Data management, Logic regression, Epidemiology

## Abstract

**Background:**

For an individual participant data (IPD) meta-analysis, multiple datasets must be transformed in a consistent format, e.g. using uniform variable names. When large numbers of datasets have to be processed, this can be a time-consuming and error-prone task. Automated or semi-automated identification of variables can help to reduce the workload and improve the data quality. For semi-automation high sensitivity in the recognition of matching variables is particularly important, because it allows creating software which for a target variable presents a choice of source variables, from which a user can choose the matching one, with only low risk of having missed a correct source variable.

**Methods:**

For each variable in a set of target variables, a number of simple rules were manually created. With logic regression, an optimal Boolean combination of these rules was searched for every target variable, using a random subset of a large database of epidemiological and clinical cohort data (construction subset). In a second subset of this database (validation subset), this optimal combination rules were validated.

**Results:**

In the construction sample, 41 target variables were allocated on average with a positive predictive value (PPV) of 34%, and a negative predictive value (NPV) of 95%. In the validation sample, PPV was 33%, whereas NPV remained at 94%. In the construction sample, PPV was 50% or less in 63% of all variables, in the validation sample in 71% of all variables.

**Conclusions:**

We demonstrated that the application of logic regression in a complex data management task in large epidemiological IPD meta-analyses is feasible. However, the performance of the algorithm is poor, which may require backup strategies.

**Electronic supplementary material:**

The online version of this article (doi:10.1186/s12911-017-0429-1) contains supplementary material, which is available to authorized users.

## Background

Today, many scientific insights are gained with meta-analyses, rather than with single studies or trials, which is illustrated with raising numbers of publications based on meta-analyses. Individual participant data (IPD) meta-analyses are far less frequent, but increasing steeply as well. Depending on the scientific question, IPD meta-analyses are superior to publication-based meta-analyses in many aspects, including the possibility to choose uniform statistical models with uniform adjustment, and—if the search is systematic—a better control of publication bias [1]. Prospectively planned pooled analyses—however optimal [1]—are still very rare, given the unproportional higher organisational effort needed.

Of course, the conduct of an IPD meta-analysis is far more laborious than a publication-based one. One large part of the workload is the harmonization of the acquired datasets. To facilitate the statistical analysis, all datasets must be transformed in a consistent format, which includes using uniform variable names and coding. In a large number of cohorts, that were planned and designed independently, the retrospective harmonization of the resulting data can become an immensely complex task [2, 3]. Furthermore, manual serial harmonization of many datasets is dull work that is prone to errors that have the potential to compromise the integrity of the meta-analysis [4]. Automated identification of variables might help to reduce the load of monotonous work, and therefore capacitates the data manager to put maximal focus on data quality [4].

The PROG-IMT project (Individual progression of carotid intima media thickness as a surrogate for vascular risk) is a large IPD meta-analysis project, with the aim to assess whether the annual change of intima media thickness (IMT, a high-resolution ultrasound measure within the carotid artery wall) is a surrogate for clinical endpoints, like myocardial infarction, stroke, or death. The project works in three stages, where a large number of datasets have been acquired, and their number is steadily growing. Details of the project plan have been published in a rationale paper [5]. The acquired datasets stem from large epidemiological population studies, from hospital cohorts and from randomized clinical trials (RCTs), each comprising between 200 and 2000 variables and between 100 and 15,000 participants. They have in common that the same set of variables is used for statistical analysis, including demographic data, vascular risk factors, and IMT. When the current project was started, we expected to acquire up to 250 individual participant datasets in heterogeneous format and coding.

In order to design a computer program that helps to reduce the workload of dataset harmonization, the first step is to find criteria to assign the correct source variable to a specific target variable in the created uniform dataset (‘allocation’). This can be attempted with simple rules, like < ‘cholesterol’ in ‘variable name’ indicates the target variable ‘total cholesterol’>; or < a median value greater than 94 indicates the target variable ‘systolic blood pressure’>. To obtain reliable performance, several of these rules have to be combined.

Logic regression is a relatively new statistical method that enables to combine simple binary rules in complex logic trees, and that provides methods to find optimal Boolean combinations [6]. As yet, this method has mostly been used in genetics [7–11] and oncology [12] to optimize complex models for disease prediction; to the best of our knowledge it hasn’t been applied to data management problems. Aim of this study was to apply logic regression techniques to the problem of assigning variables, as explained above, and to validate the performance of this approach, using data from the PROG-IMT project.

## Methods

**Fig. 1:**
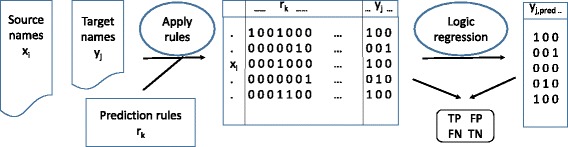
Fictitious example of a logic tree combining allocation rules

The PROG-IMT project is involved in using datasets from population-based epidemiologic studies, from risk populations and from RCTs. At the time these analyses were started, 34 datasets were available that were already manually harmonized. These were randomly (1:1) assigned to a construction subset, or a validation subset (Table 1). All these datasets include many variables; some of those correspond to predefined target variables, which are needed for the statistical analysis of the main project. This set of target variables is shown in Table 2. The overall algorithm followed is shown graphically in Fig. 1.

**Table 1:** Datasets used for construction and validation

Acronym or designation	Study name	Study type	Number of variables	Number of participants	Use
AIR	Atherosclerosis and Insulin Resistance study	general population	136	435	Construction
ARIC	Atherosclerosis risk in communities	general population	10108	15042	Validation
BCAPS	Beta-blocker Cholesterol-lowering Asymptomatic Plaque Study	RCT	134	1544	Validation
BHS	Bogalusa Heart Study	general population	1220	1986	Construction
BKRE	Konyang University Hospital CIMT Registry	RCT	109	205	Validation
Bruneck	Bruneck Study	general population	141	821	Validation
CAPS	Carotid Atherosclerosis Progression Study	general population	692	6972	Construction
CCCC	Chin-Shan Community Cardiovascular Cohort Study	general population	110	3603	Construction
CHS	Cardiovascular Health Study	general population	1426	5901	Construction
CIMT_TIME	CIMT TIME Project	risk population	144	671	Validation
CMCS-Beijing	Chinese Multi-provincial Cohort Study-Beijing	general population	141	1324	Construction
CREED	Cardiovascular Risk Extended Evaluation in Dialysis patients	risk population	53	138	Construction
DIWA	Diabetes, Impaired glucose tolerance in Women and Atherosclerosis	general population	129	644	Validation
EAS	Edinburgh Artery Study	general population	74	1593	Construction
Ekart et al.	None	risk population	102	54	Construction
EPICARDIAN	EPIdemiología CARDIovascular en los ANcianos, Cardiovascular Epidemiology in the Elderly in Spain	general population	76	446	Construction
EVA	Etude du Vieillissement Arteriel	general population	212	1135	Validation
HD-IMT	Carotid ultrasonographic parameters as markers of atherogenesis and mortality rate in patients on hemodialysis	risk population	130	85	Validation
HOORN	The Hoorn Study	general population	128	3103	Construction
IMPROVE	Carotid Intima Media Thickness and IMT-Progression as Predictors of Vascular Events in a High Risk European Population	risk population	103	3703	Construction
INVADE	Interventionsprojekt zerebrovaskuläre Erkrankungen und Demenz im Landkreis Ebersberg	general population	1581	3365	Validation
Kato et al.	None	risk population	131	284	Validation
KIHD	Kuopio Ischemic Heart Disease Risk Factor Study	general population	151	1399	Construction
Landecho et al.	None	risk population	69	248	Validation
Niguarda	Niguarda-Monzino Study	risk population	88	1564	Construction
NOMAS/INVEST	Northern Manhattan Study	general population	334	857	Validation
OSACA	Osaca Follow-Up Study for Carotid Atherosclerosis	risk population	108	291	Construction
Papagianni et al.	None	risk population	73	84	Construction
PIVUS	Prospective Investigation of the Vasculature in Uppsala Seniors	general population	98	1017	Validation
PLIC	Progression of Lesions in the Intima of the Carotid	general population	264	2607	Validation
RIAS	Resistive Index in AtheroSclerosis	risk population	67	158	Construction
Rotterdam	Rotterdam Study	general population	34	7983	Validation
SAPHIR	Salzburg Atherosclerosis Prevention program in subjects at High Individual Risk	general population	141	3127	Validation
SHIP	Study of Health in Pomerania	general population	320	4308	Construction

**Table 2:** Sensitivity, specificity, PPV and NPV of the optimal Boolean combinations in the construction and in the validation sample

Variable	Unit	Construction sample				Validation sample			
		Sensitivity	Specificity	PPV	NPV	Sensitivity	Specificity	PPV	NPV
Age	years	1	0.997549	0.78	1	0.956522	0.994545	0.511628	0.999739
BMI	kg/m^2^	0.973684	0.999777	0.973684	0.999777	0.454545	0.998958	0.789474	0.99533
Urea	mg/dl	1	0.78333	0.004065	1	1	0.822693	0.005797	1
Cholesterol	mg/dl	0.913043	0.999778	0.954545	0.999556	0.588235	1	1	0.998188
Cholesterol SI	mmol/l	0.956522	0.463366	0.00902	0.999521	0.375	0.443609	0.002788	0.994189
Creatinine	mg/dl	0.947368	0.727595	0.014446	0.999695	0.777778	0.825311	0.010264	0.999373
Diabetes	-	1	0.005797	0.009331	1	0.757576	0.015625	0.00657	0.882353
Education	-	0.866667	0.499778	0.005727	0.999114	0.888889	0.508799	0.004197	0.999492
Ethnicity	-	0.916667	0.998671	0.647059	0.999778	0.625	0.998706	0.5	0.999223
Event date	-	0.771084	0.455221	0.025755	0.990695	0.580645	0.407407	0.02354	0.975301
Fasting glucose	mg/dl	0.954545	0.980022	0.189189	0.999774	0.5625	0.994555	0.3	0.998179
Fasting glucose SI	mmol/l	1	0.015289	0.00314	1	0.866667	0.020477	0.003428	0.975309
Fibrinogen	mg/dl	0.928571	0.515622	0.005912	0.99957	1	0.556648	0.004067	1
Hemoglobin	g/dl	0.923077	0.145104	0.0031	0.998476	0.875	0.124191	0.002064	0.997921
Hemoglobin SI	g/l	1	0.024326	0.001132	1	0	0.047065	0	0.968085
Hba1c	%	0.944444	0.999113	0.809524	0.999778	0.666667	1	1	0.998965
HDL cholesterol	mg/dl	0.35	0.999334	0.7	0.997122	0.1875	0.998704	0.375	0.996636
HDL cholesterol SI	mmol/l	0.863636	0.978246	0.162393	0.99932	0.705882	0.977178	0.12	0.998675
History of CVD	-	0.611111	0.98368	0.311321	0.99525	0.348485	0.967923	0.277108	0.976801
Hs-CRP	mg/l	0.875	0.99823	0.466667	0.999778	1	0.998702	0.807692	1
Hypertension	-	0.941176	0.971511	0.2	0.999542	0.965517	0.961238	0.158192	0.999729
Intima Media Thickness (IMT)	0.1 mm	0.73494	0	0.013541	0	0.99115	0	0.057866	0
Intima Media Thickness (IMT) SI	mm	0.354911	0.989703	0.791045	0.933195	0.070588	0.969044	0.138462	0.936682
Arterial diameter	mm	0.685393	0.989184	0.559633	0.993662	0.509259	0.998406	0.901639	0.986097
Income	-	1	0	0.000884	0	1	0	0.000516	0
LDL cholesterol	mg/dl	0	1	0	0.995582	0	1	0	0.996902
LDL cholesterol	mmol/l	0.73913	0.992451	0.333333	0.99866	0.5	0.996368	0.391304	0.997662
Leukocytes	1/μl	0	0.993803	0	0.998	0	0.997411	0	0.997152
Dyslipidemia	-	0.923077	0.999335	0.8	0.999778	0.416667	0.998446	0.454545	0.998187
Antidiabetic medication	-	0.761905	0.997074	0.831169	0.995506	0.571429	0.998688	0.878049	0.992954
Antihypertensive medication	-	0.989362	0.591699	0.04887	0.999619	0.886364	0.607057	0.073724	0.993438
Lipid-lowering medication	-	0.987179	0.510227	0.034131	0.99956	0.982143	0.5093	0.028527	0.999486
Nicotine consumption	Pack years	0.8	1	1	0.998891	0.714286	0.999482	0.833333	0.998964
Carotid plaque	-	0.613636	0.999108	0.870968	0.996219	0.953757	1	1	0.997843
Diastolic blood pressure	mmHg	0.965116	0.985364	0.560811	0.999315	0.764706	0.983255	0.378641	0.996817
Systolic blood pressure	mmHg	0.674419	0.999775	0.983051	0.993733	0.510638	0.996602	0.648649	0.994004
Socioeconomic status	-	1	0.076514	0.004304	1	0.888889	0.072205	0.002227	0.996429
Sex	-	0.875	0.999334	0.875	0.999334	0.6875	0.998444	0.647059	0.998703
Smoking status	-	0.972603	0.20229	0.019592	0.997785	0.75	0.26145	0.013632	0.987154
Triglycerides	mg/dl	0.969697	0.136849	0.008182	0.998377	0	0.077201	0	1
Ultrasound date	-	0	0.999553	0	0.988729	0	1	0.988897	1
Average		0.799584	0.707405	0.34172	0.947871	0.619019	0.710383	0.325339	0.941999

In a first step, a set of simple rules was manually created (four to 41) for every target variable, by an epidemiologist experienced in the handling of data of this type. These rules are described in Additional file 1: Table S1. These rules included conditions on the variable name, the variable label, variable type (number, date or string), scale level (ratio, ordinal or nominal, dichotomous nominal); in nominal or ordinal variables the number of values and the proportion of the most frequent value; and in ratio variables the median and the interquartile range.

For rules that involved a cutoff value (eg. median greater than 44), this cutoff was optimized with ROC analysis, with the aim to maximize the expression ‘sensitivity + specificity’. For every target variable, logic regression models were created by Boolean combination of the specific rules, or a subset of these. To find an optimal Boolean combination of rules (example in Fig. 1), we applied the ‘simulated annealing’ algorithm [4].

Simulated annealing is a generic optimization procedure commonly used to optimize non-convex optimization problems. It presupposes that an application specific score or evaluation or loss function has been defined which assigns a penalty to each state of a system. Simulated annealing then iteratively perturbs the system using applications specific basic operations, in this case tree pruning manipulations as mentioned below, with the aim of reducing the score value of the perturbed state. The perturbations are chosen in a random way with state transition probabilities changing in the course of the iteration. This lowering of transition probabilities is the analogue of lowering of temperature in random motion in physical science and is the basic mechanism in simulated annealing to reduce the danger of missing the global optima, while at the same time allowing for convergence of the iteration. In the current work transition probabilities were systematically reduced from 0.1 to 0.0001. When using simulated annealing for logic regression in the context of identifying source variable names, the states of the system are logical expressions, like for example (R_1_ v R_2_) ʌ R_3_ that assign a true or false value to candidate variable name based on the rules R_1_, R_2_, R_3_. The evaluation function was a weighted least squares function of the type *SWS*_*res*_ *= Σ w*_*i*_ *(y*_*i*_ *– y*_*i,pred*_ *)* ^*2*^, which in the case of classification, where *y*_*i*_ and *y*_*i,pred*_ are 0 or 1, is just a weighted misclassification count. In order to increase sensitivity without undue loss of specificity, much higher weight was given to the positives (0.9995, opposed to 0.0005 to the negatives), thus compensating the much higher number of negatives, and the basic operations are changes in the logical expression like “alternating leaves”, “alternating operators”, “growing a branch”, “pruning a branch”, “splitting a leaf” or “deleting a leaf”. The names of these operations are better understood, when visualizing a logical expression as a tree.

In order to understand the dependency of sensitivity and specificity on the tuning parameters of the annealing algorithm a factor analysis was performed. Two methods were used, classification and logistic regression, four different weights for the negatives, 5*10^-4^, 5*10^-3^, 5*10^-2^, and 5*10^-1^, two tree sizes 5 and 10 and two values namely 4 and 8 were used for the minimum number of cases for which the tree needs to be 1. A 2^3^ x 4 hybrid factorial design was performed. This yielded 32 runs for sensitivity and specificity and allowed finding interactions between the factors.

**Fig. 2:**
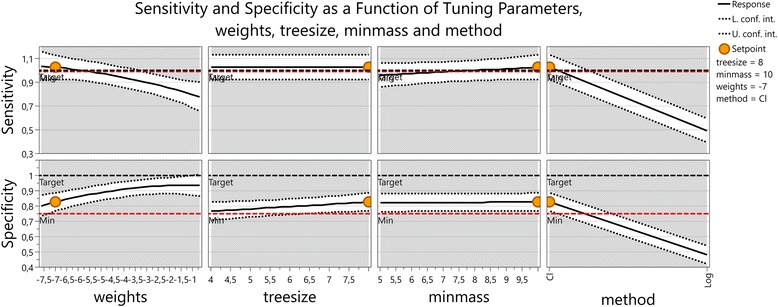
Sensitivity and specificity as a function of tuning parameters, weights, treesize, minmass and method. At the set point weights = exp(-7), treesize = 8, minmass = 10 for the classification method, the dependency of sensitivity and specificity upon these tuning parameters can be read off this multiple one dimensional plot. On the x-axis in the left most plot, weights are shown as natural logarithm of the actual values that effectively vary from 0.0005 = exp(-7.6) to 0.5 = exp(-0.7)

**Fig. 3:**
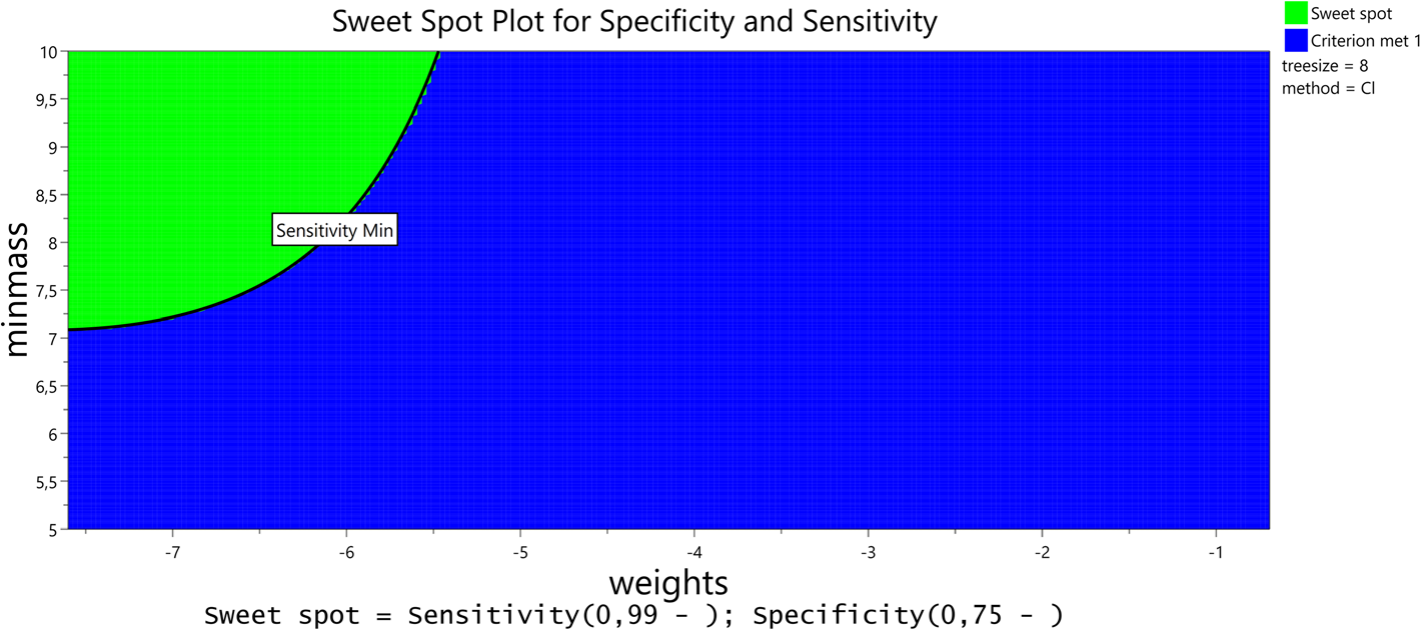
Sweetspot plot for sensitivity and specificity. The same information as in Fig. 2 as a two dimensional Contour Plot (Sweet Spot Plot) for Specificity and Sensitivity. For low values of weights and high values of minmass, treesize = 8 and the classification method, sensitivity can be raised above 99% without lowering specificity below 75%. On the x-axis, weights are again shown as natural logarithm of the actual values

An optimization with the aim of maximizing sensitivity (low limit 99%) and specificity (low limit 75%) followed by dynamic profiling gave the result that direct classification is better than logistic regression and that due to the high interaction between the weights and the classification method, low weights are important to achieve high sensitivity. The loss in specificity that results from lowering the weights is less important than the gain in sensitivity (Figs. 2 and 3).

To find optimal combinations of rules for every target variable we used the training subset of datasets. Logic regression was applied in several models, where different configuration parameters, such as the weight of cases (matching variables) and controls (non-matching variables), and the link function itself (classification or logistic model), were varied.

After optimal configuration parameters were found, the stability of the method was tested using cross-validation: each 10% of the data were predicted from models derived from the remaining 90% of data in turn. As it is a typical characteristic of logic regression that different source data result in qualitatively very different logic trees, these models couldn’t be compared on the procedural level. Therefore we compared the resulting model quality in terms of sensitivity and specificity to detect a specific target variable.

The best model was fixed, and used to predict the correct assignment of variables in the validation sample. The resulting precision in the validation data was assessed using sensitivity, specificity, positive and negative predictive values. In the context of the present study, sensitivity of a target variable is the portion of matching source variables that are correctly identified. Positive predictive value (PPV) is the portion of identified source variables for which the identification is correct. Correspondingly, specificity is the portion of non-matching source variables that are identified as such and negative predictive value (NPV) is the portion of negatively identified source variables for which this identification is correct.

The source data were prepared with SAS version 9.3 (The SAS Institute, Cary, USA) and stored into a.csv file format. For the data handling and logic regression we wrote programs within C#, using R and R.NET libraries, including those from the R software package developed by Ingo Ruczinski, Charles Kooperberg, and Michael LeBlanc at the Fred Hutchinson Cancer Research Center in Seattle (CRAN package version 3). The design for the optimization of tuning parameters and the optimization were done with MODDE Pro version 11 (mks Data Analytic Solutions, Umea, Sweden).

## Results

As expected from a classification algorithm using a tree based method the logic trees themselves were quite different among different cross validation runs and due to the character of the simulated annealing algorithm even for repeated runs with the same input data. However the measured sensitivity and specificity of different runs of the algorithm were quite stable and allowed for reliable comparisons. The complete best models for every target variable are shown in Additional file 1: Table S1. Table 2 shows the performance parameters of these best models. In columns 3–6, the results in the construction sample are displayed. Sensitivity was on average reasonable high (0.80), as was the specificity (0.70). The PPV was overall poor (on average 0.34), NPV was good (average 0.95). In columns 7–10 we showed the results of independent validation (in the validation sample). Here, sensitivity was considerable less (0.62), but specificity was comparable (0.71), just as PPV (0.33) and NPV (0.94).

## Discussion

The performance was quite heterogeneous: in some target variables, sensitivity, specificity, PPV and NPV were very high (e.g. age, antidiabetic medication). However, many other variables showed PPV that was far too low to be useful even in the construction sample. For the intended use within a computer program to support the data manager, the performance of the models seemed reasonable at the first glance, in terms of sensitivity. However, in order to determine the correct source variable for a given target variable, the most important quality indicator is PPV, which is the portion of identified source variables for which the identification is correct. When the PPV is considered, the performance of the algorithm was much worse. In fact, the majority of variable had PPV values of 50% or less (63% in the construction sample, 71% in the validation sample). With failure rates as high as observed in the validation sample, a fictitious computer program would have to give a list of several candidate variables rather than a single result, for each target variable. Furthermore, an escape pathway would have to be implemented for the case that the true target variable was not on the list suggested by the program. However, even if the algorithm can only give a ‘first guess’ which is correct in 50%, it may reduce the workload of the data manager by nearly half.

Still, from a methodologic perspective, it is remarkable that a tree based classification method based on a random process such as the ‘simulated annealing’ behaves in a reproducible fashion, on the result level, i.e. regarding quality characteristics such as sensitivity and specificity. The overall performance of the optimized logic regression models in the validation sample, compared to the construction sample, is quite similar to linear regression prediction models, for example. A finding that is worth noticing is that our attempts to optimize for sensitivity were counteracted by the models. For the intended use, sensitivity is more important than specificity, and PPV is more important than NPV, as a human data manager has more difficulty reviewing many variables than a short list of candidates, as long as he or she can rely on the fact that the target variable is on this short list. Therefore, we undertook efforts to optimize the evaluation function of the algorithm for high sensitivity and high PPV. In the construction sample this worked nicely by weighting the positives by 0.9995 against 0.0005 for the negatives, i.e. a factor of 1999, for the negatives. This improved sensitivity from 0.976 (0.995 against 0.005, i.e. 199) to 0.99948, while reducing specificity from 0.87 to 0.78. Interestingly enough, as can be verified in Table 2, the same models with the same weighting turned out to be more specific than sensitive in the validation sample.

As reflected by the increase of the number of meta-analyses over time, many insights may be gained with large collaborative projects collating data from many participating cohorts in the future [13]. Although, from the methodological point of view, the best form of meta-analyses are most likely prospectively planned pooled analyses [1, 13], such projects are still rare. This may be due to the immense efforts and high volumes of funding they require; furthermore such enterprises take many years or even decades to complete. So in the near and intermediate future, we will most likely increasingly face the ‘second best option’ [1]: IPD meta-analyses that require retrospective harmonization of data [14].. Whereas some meta-analyses have developed impressively professional structures and algorithms [2–4] and the overall quality of IPD meta-analyses has improved over the last decade [15], there still remains scope for improving their processes and statistical methods [14, 15].

To date, the aspects that are discussed in published literature include mostly statistical modelling [15–19], sometimes screening [15, 16], and rarely the process of harmonization of data [2–4]. Fortier et al. [2] and Doiron et al. [3] both describe detailed algorithms for the harmonization of heterogeneous data including manual allocation of target variables. Bosch-Capblanc [4] suggested a computer program with a three-stage algorithm to detect the matching source variable for each given target variable. Compared to our algorithm, the identification criteria are less refined, and it includes alternative ways of allocating if the primary identification criteria failed. To the best of our knowledge, no publication so far has refined the allocations procedures to the extent we have. As the Bosch-Capblanc algorithm [4] focused more on the actual handling of the data, a combination of his algorithm with our allocation procedure may yield excellent results, which remains to be tested.

However, the process shown here needs relevant manual preparations before an automated or semi-automated process can start, e.g. the manual definition of target-variable rules. This preparatory work is depending on the number of target variables, whereas the work saved by automating depends on the number of datasets processed. These benchmark data have to be weighted carefully to decide whether this approach is economic. Most likely, it will be economic when many datasets are processed, and few target variables are needed. If the rule definitions might be automated, too, this might facilitate the application considerably, improve reproducibility and reduce investigator bias.

## Conclusions

With the current work we demonstrated that it is in principle possible to use logic regression models with the automated ‘simulated annealing’ algorithm for the task of allocating variables in large datasets to specific target variables. With the performance shown in the present example, however, it would be necessary to introduce precautions in the design of a computer program, to avoid missing the true matching source variable. Such precautions may include the program suggesting a list of candidate variables rather than a single matching variable, and the option of an exit path with manual allocation. In any case, the development effort for algorithm, optimal models and a computer program is very high, and may only amortize if several hundred datasets have to be handled.

## Additional file

Additional file 1: Table S1. Rules for specific target variables and their best Boolean combination. **Table S2.** Program parameters. List of Members of the PROG-IMT Study group. (DOC 744 kb)

## Abbreviations

IMT: Intima media thickness
IPD: Individual participant data
NPV: Negative predictive value
PPV: Positive predictive value
PROG-IMT: The ‘Individual progression of carotid intima media thickness as a surrogate for vascular risk’ project
RCT: Randomized controlled trial
ROC: Receiver operating characteristic
