# Supplementary material for: Automatic identification of variables in epidemiological datasets using logic regression
Source: BMC Med Inform Decis Mak. 2017 Apr 13;17:40. doi: 10.1186/s12911-017-0429-1 (PMC5390441; doi:10.1186/s12911-017-0429-1)
Supplement: Additional file 1: Table S1. — Rules for specific target variables and their best Boolean combination. Table S2. Program parameters. List of Members of the PROG-IMT Study group. (DOC 744 kb) [file 12911_2017_429_MOESM1_ESM.doc]

# Additional files

Table S1: Rules for specific target variables and their best Boolean combination

| **Target variable** | **Predefined rules in C# syntax** | **Predefined rules in plain text form** | **Boolean combination in the best model** |
| --- | --- | --- | --- |
| Age | | X1 : index(label,"age")>0 | | --- | | X2 : index(label,"alter")>0 | | X3 : index(name,"age")>0 | | X4 : index(name,"alter")>0 | | X5 : var_iqr < 27 | | X6 : var_iqr > 3,9 | | X7 : var_med < 79 | | X8 : var_med > 15,23 | | X9 : (var_s = 1) > 0 | | | X1 : "age" in label | | --- | | X2 : "alter" in label | | X3 : "age" in name | | X4 : "alter" in name | | X5 : interquartile range < 27 | | X6 : interquartile range > 3,9 | | X7 : median < 79 | | X8 : median > 15,23 | | X9 : scale level is ratio | | (((X6 and X3) or (X7 and X3)) and ((X2 or X8) and (X1 or X2))) |
| BMI | | X1 : index(label,"bmi")>0 | | --- | | X2 : index(label,"body mass index")>0 | | X3 : index(label,"body")>0 | | X4 : index(label,"kg/m")>0 | | X5 : index(name,"bmi")>0 | | X6 : index(name,"body mass index")>0 | | X7 : index(name,"body")>0 | | X8 : var_iqr < 7,484 | | X9 : var_iqr > 3,12 | | X10 : var_med < 28,84 | | X11 : var_med > 22,3 | | X12 : (var_s = 1) > 0 | | | X1 : “bmi” in label | | --- | | X2 : "body mass index” in label | | X3 : “body” in label | | X4 : “kg/m” in label | | X5 : “bmi” in name | | X6 : “body mass index” in name | | X7 : “body” in name | | X8 : interquartile range < 7,484 | | X9 : interquartile range > 3,12 | | X10 : median < 28,84 | | X11 : median > 22,3 | | X12 : scale level is ratio | | (((X5 and X11) or (X1 and X8)) and ((X1 and X5) or (X12 or (not X10)))) |
| Urea | | X1 : (var_s = 1) > 0 | | --- | | X2 : index(label,"bun")>0 | | X3 : index(label,"mg/dl")>0 | | X4 : index(label,"urea")>0 | | X5 : index(name,"bun")>0 | | X6 : index(name,"urea")>0 | | X7 : var_iqr < 0,07 | | X8 : var_iqr > 0,6 | | X9 : var_med < 0,33 | | X10 : var_med > 0,25 | | | X1 : scale level is ratio | | --- | | X2 : “bun” in label | | X3 : "mg/dl” in label | | X4 : "urea" in label | | X5 : "bun" in name | | X6 : "urea" in name | | X7 : interquartile range < 0,07 | | X8 : interquartile range > 0,6 | | X9 : median < 0,33 | | X10 : median > 0,25 | | (((not X8) and (not X7)) and ((not X9) and (not X3))) |
| Cholesterol | | X1 : index(label,"chol")>0 | | --- | | X2 : index(label,"chol")>0 & index(label,"hdl") = 0 | | X3 : index(label,"chol")>0 & index(label,"hdl") = 0 & index(label,"ldl") = 0 | | X4 : index(label,"chol")>0 & index(label,"ldl") = 0 | | X5 : index(label,"gesamt")>0 & index(label,"chol") > 0 | | X6 : index(label,"mg/dl")>0 | | X7 : index(label,"tc")>0 | | X8 : index(label,"total")>0 & index(label,"chol") > 0 | | X9 : index(name,"chol")>0 | | X10 : index(name,"chol")>0 & index(name,"hdl") = 0 | | X11 : index(name,"chol")>0 & index(name,"hdl") = 0 & index(name,"ldl") = 0 | | X12 : index(name,"chol")>0 & index(name,"ldl") = 0 | | X13 : index(name,"gesamt")>0 & index(name,"chol") > 0 | | X14 : index(name,"tc")>0 | | X15 : index(name,"total")>0 & index(name,"chol") > 0 | | X16 : var_iqr < 65 | | X17 : var_iqr > 36,5 | | X18 : var_med < 228,5 | | X19 : var_med > 166 | | X20 : (var_s = 1) > 0 | | | X1 : "chol" in label | | --- | | X2 : "chol" in label and "hdl" not in label | | X3 : "chol" in label and "hdl" and "ldl"  not in label | | X4 : "chol" in label and "ldl" not in label | | X5 : "gesamt" and "chol“ in label | | X6 : "mg/dl" in label | | X7 : "tc" in label | | X8 : "total" and "chol" in label | | X9 : "chol" in name | | X10 : "chol" in name and and "hdl"  not in name | | X11 : "chol" and "hdl" and “ldl” in name | | X12 : "chol" and "ldl" in name | | X13 : "gesamt" and "chol“ in name | | X14 : "tc" in name | | X15 : "total" and "chol“ in name | | X16 : interquartile range < 65 | | X17 : interquartile range > 36,5 | | X18 : median < 228,5 | | X19 : median > 166 | | X20 : scale level is ratio | | (((X18 and X19) or X5) and (X17 or X9)) |
| Cholesterol SI | | X1 : index(label,"chol")>0 | | --- | | X2 : index(label,"chol")>0 & index(label,"hdl") = 0 | | X3 : index(label,"chol")>0 & index(label,"hdl") = 0 & index(label,"ldl") = 0 | | X4 : index(label,"chol")>0 & index(label,"ldl") = 0 | | X5 : index(label,"chol")>0 & index(label,"mmol/l") > 0 | | X6 : index(label,"gesamt")>0 & index(label,"chol") > 0 | | X7 : index(label,"mmol/l") > 0 | | X8 : index(label,"tc")>0 | | X9 : index(label,"total")>0 & index(label,"chol") > 0 | | X10 : index(name,"chol")>0 | | X11 : index(name,"chol")>0 & index(name,"hdl") = 0 | | X12 : index(name,"chol")>0 & index(name,"hdl") = 0 & index(name,"ldl") = 0 | | X13 : index(name,"chol")>0 & index(name,"ldl") = 0 | | X14 : index(name,"gesamt")>0 & index(name,"chol") > 0 | | X15 : index(name,"tc")>0 | | X16 : index(name,"total")>0 & index(name,"chol") > 0 | | X17 : var_iqr < 1,7 | | X18 : var_iqr > 1,13 | | X19 : var_med < 6,97 | | X20 : var_med > 4,1999 | | X21 : (var_s = 1) > 0 | | | X1 : "chol" in label | | --- | | X2 : "chol" in label and "hdl" not in label | | X3 : "chol" in label and "hdl" and "ldl"  not in label | | X4 : "chol" in label and "ldl" not in label | | X5 : "chol" and "mmol/l“ in label | | X6 : "gesamt" and "chol“ in label | | X7 : "mmol/l" in label | | X8 : "tc" in label | | X9 : "total" and "chol" in name | | X10 : "chol" in name | | X11 : "chol" in name and "hdl" not in name | | X12 : "chol" in name and "ldl" and "hdl”  not in name | | X13 : "chol“ in name and "ldl“ not in name | | X14 : "gesamt" and "chol“ in name | | X15 : "tc“ in name | | X16 : "total" and "chol“ in name | | X17 : interquartile range < 1,7 | | X18 : interquartile range > 1,13 | | X19 : median <6,97 | | X20 : median > 4.1999 | | X21 : scale level is ratio | | (((X20 and X19) and (X17 and X18)) or ((X12 and (not X16)) or (X15 and (not X8)))) |
| Creatinine | | X1 : (var_s = 1) > 0 | | --- | | X2 : index(label,"crea")>0 | | X3 : index(label,"crn")>0 | | X4 : index(label,"krea")>0 | | X5 : index(name,"crea")>0 | | X6 : index(name,"crn")>0 | | X7 : index(name,"krea")>0 | | X8 : var_iqr < 20 | | X9 : var_iqr > 0,1988 | | X10 : var_med < 101 | | X11 : var_med > 0,7933 | | | X1 : scale level is ratio | | --- | | X2 : "crea" in label | | X3 : "crn" in label | | X4 : "krea" in label | | X5 : "crea" in label | | X6 : "crn" in name | | X7 : "krea" in name | | X8 : interquartile range < 20 | | X9 : interquartile range > 0,1988 | | X10 : median < 101 | | X11 : median > 0,7933 | | (((X1 and X9) and (X11 or X2)) and ((X10 and X8) or (X9 and X2))) |
| Diabetes | | X1 : index(label,"diab")>0 | | --- | | X2 : index(label,"diabet")>0 | | X3 : index(label,"diabetes")>0 | | X4 : index(label,"dm")>0 | | X5 : index(label,"hypogly")>0 | | X6 : index(name,"diab")>0 | | X7 : index(name,"diabet")>0 | | X8 : index(name,"diabetes")>0 | | X9 : index(name,"dm")>0 | | X10 : index(name,"hypogly")>0 | | X11 : var_nr < 4 | | X12 : var_nr > 1 | | X13 : var_pr < 97,2541 | | X14 : var_pr > 37,1343 | | X15 : (var_s = 3) > 0 | | | X1 : "diab" in label | | --- | | X2 : "diabet" in label | | X3 : "diabetes" in label | | X4 : "dm" in label | | X5 : "hypogly" in label | | X6 : "diab" in name | | X7 : "diabet" in name | | X8 : "diabetes" in name | | X9 : "dm" in name | | X10 : "hypogly" in name | | X11 : number of values < 4 | | X12 : number of values > 1 | | X13 : proportion of most  frequent category < 97,2541 | | X14 : proportion of most  frequent category > 37,1343 | | X15 : scale level is dichotomous nominal | | (((X13 and X12) and (X14 and X15)) or ((X2 or X4) and (X14 or X15))) |
| Education | | X1 : index(label,"bild")>0 | | --- | | X2 : index(label,"grad")>0 | | X3 : index(label,"school")>0 | | X4 : index(label,"schul")>0 | | X5 : index(name,"bild")>0 | | X6 : index(name,"edu")>0 | | X7 : index(name,"grad")>0 | | X8 : index(name,"school")>0 | | X9 : index(name,"schul")>0 | | X10 : var_nr < 10 | | X11 : var_nr > 1 | | X12 : var_pr < 71,0843 | | X13 : var_pr > 12,0996 | | X14 : (var_s = 3) > 0 | | | X1 : "bild" in label | | --- | | X2 : "grad" in label | | X3 : "school" in label | | X4 : "schul" in label | | X5 : "bild" in name | | X6 : "edu" in name | | X7 : "grad" in name | | X8 : "school" in name | | X9 : "schul" in name | | X10 : number of values < 10 | | X11 : number of values > 1 | | X12 : proportion of most frequent caregory < 71,0843 | | X13 : proportion of most frequent caregory > 12,0996 | | X14 : scale level is dichotomous nominal | | (((X8 and X4) or (X9 or X7)) or ((X1 and X2) or (X6 or X7))) |
| Ethnicity | | X1 : index(label,"afri")>0 | | --- | | X2 : index(label,"black")>0 | | X3 : index(label,"cauca")>0 | | X4 : index(label,"ethnicity")>0 | | X5 : index(label,"hispa")>0 | | X6 : index(label,"race")>0 | | X7 : index(name,"eth")>0 | | X8 : index(name,"rac")>0 | | X9 : index(name,"race")>0 | | X10 : var_nr < 1 | | X11 : var_nr > 3 | | X12 : var_pr < 90,4093 | | X13 : var_pr > 69,7648 | | X14 : (var_s = 3) > 0 | | | X1 : "afri" in label | | --- | | X2 : "black" in label | | X3 : "cauca" in label | | X4 : "ethnicity" in label | | X5 : "hispa" in label | | X6 : "race" in label | | X7 : "eth" in name | | X8 : "rac" in name | | X9 : "race" in name | | X10 : number of values < 1 | | X11 : number of values > 3 | | X12 : proportion of most frequent caregory < 90,4093 | | X13 : proportion of most frequent caregory > 69,7648 | | X14 : scale level is dichotomous nominal | | ((X6 or (X8 and X11)) or X7) |
| Event date | | X1 : var_iqr < 110210 | | --- | | X2 : var_iqr > 349 | | X3 : var_med < 20010226 | | X4 : var_med > 719 | | X5 : (var_s = 1) > 0 | | | X1 : interquartile range < 110210 | | --- | | X2 : interquartile range > 349 | | X3 : median < 20010226 | | X4 : median > 719 | | X5 : scale level is ratio | | ((((not X3) or (not X2)) and (X1 and (not X3))) or ((not X2) or (X4 and X3))) |
| Fasting glucose | | X1 : index(label,"fast")>0 & index(label,"glu") > 0 | | --- | | X2 : index(label,"glu")>0 | | X3 : index(label,"gluc")>0 | | X4 : index(label,"mg/dl")>0 | | X5 : index(name,"fast")>0 & index(name,"glu") > 0 | | X6 : index(name,"glu")>0 | | X7 : index(name,"gluc")>0 | | X8 : var_iqr < 2,1 | | X9 : var_iqr > 0,6 | | X10 : var_med < 6 | | X11 : var_med > 4,23 | | X12 : (var_s = 1) > 0 | | | X1 : "fast" in label and "glu" in label | | --- | | X2 : "glu" in label | | X3 : "gluc" in label | | X4 : "mg/dl" in label | | X5 : "fast" in name and "glu" in name | | X6 : "glu" in name | | X7 : "gluc" in name | | X8 : interquartile range < 2,1 | | X9 : interquartile range > 0,6 | | X10 : median < 6 | | X11 : median > 4,23 | | X12 : scale level is ratio | | (((X8 or (not X12)) and X1) or ((X11 and X10) and (X8 or X2))) |
| Fasting glucose SI | | X1 : index(label,"fast")>0 & index(label,"glu") > 0 | | --- | | X2 : index(label,"glu")>0 | | X3 : index(label,"gluc")>0 | | X4 : index(label,"mmol/l")>0 | | X5 : index(name,"fast")>0 & index(name,"glu") > 0 | | X6 : index(name,"glu")>0 | | X7 : index(name,"gluc")>0 | | X8 : var_iqr < 21 | | X9 : var_iqr > 12,6666 | | X10 : var_med < 108 | | X11 : var_med > 4,23 | | X12 : (var_s = 1) > 0 | | | X1 : "fast" in label and "glu" in label | | --- | | X2 : "glu" in label | | X3 : "gluc" in label | | X4 : "mmol/l" in label | | X5 : "fast" in name and "glu" in name | | X6 : "glu" in name | | X7 : "gluc" in name | | X8 : interquartile range < 21 | | X9 : interquartile range > 12,6666 | | X10 : median < 108 | | X11 : median > 4,23 | | X12 : scale level is ratio | | (((X6 and (not X1)) and X9) or ((X9 and X10) and (X8 and X11))) |
| Fibrinogen | | X1 : index(label,"clauss")>0 | | --- | | X2 : index(label,"fib")>0 | | X3 : index(label,"fibri")>0 | | X4 : index(name,"clauss")>0 | | X5 : index(name,"fib")>0 | | X6 : index(name,"fibri")>0 | | X7 : var_iqr < 109 | | X8 : var_iqr > 0,72 | | X9 : var_med < 355 | | X10 : var_med > 2,4 | | X11 : (var_s = 1) > 0 | | | X1 : "clauss" in label | | --- | | X2 : "fib" in label | | X3 : "fibri" in label | | X4 : "clauss" in name | | X5 : "fib" in name | | X6 : "fibri" in name | | X7 : interquartile range < 109 | | X8 : interquartile range > 0,72 | | X9 : median < 355 | | X10 : median > 2,4 | | X11 : scale level is ratio | | (((X2 and X11) or (X3 and X5)) or (((not X10) or (not X2)) and (X1 and (not X8)))) |
| Hemoglobin | | X1 : index(label,"globin")>0 | | --- | | X2 : index(label,"hb")>0 | | X3 : index(label,"hemo")>0 | | X4 : index(label,"hemoglobin")>0 | | X5 : index(label,"mg/dl")>0 | | X6 : index(name,"globin")>0 | | X7 : index(name,"hb")>0 | | X8 : index(name,"hemo")>0 | | X9 : index(name,"hemoglobin")>0 | | X10 : var_iqr < 14,5 | | X11 : var_iqr > 1,485 | | X12 : var_med < 147,5 | | X13 : var_med > 13,5962 | | X14 : (var_s = 1) > 0 | | | X1 : "globin" in label | | --- | | X2 : "hb" in label | | X3 : "hemo" in label | | X4 : "hemoglobin" in label | | X5 : "mg/dl" in label | | X6 : "globin" in name | | X7 : "hb" in name | | X8 : "hemo" in name | | X9 : "hemoglobin" in name | | X10 : interquartile range < 14,5 | | X11 : interquartile range > 1,485 | | X12 : median < 147,5 | | X13 : median > 13,5962 | | X14 : scale level is ratio | | (((X12 or X10) and (not X2)) and ((X13 and X11) and ((not X5) or X4))) |
| Hemoglobin SI | | X1 : index(label,"globin")>0 | | --- | | X2 : index(label,"hb")>0 | | X3 : index(label,"hemo")>0 | | X4 : index(label,"hemoglobin")>0 | | X5 : index(label,"mmol/l")>0 | | X6 : index(name,"globin")>0 | | X7 : index(name,"hb")>0 | | X8 : index(name,"hemo")>0 | | X9 : index(name,"hemoglobin")>0 | | X10 : var_iqr < 29,59 | | X11 : var_iqr > 2,502 | | X12 : var_med < 10,4 | | X13 : var_med > 3,3 | | X14 : (var_s = 1) > 0 | | | X1 : "globin" in label | | --- | | X2 : "hb" in label | | X3 : "hemo" in label | | X4 : "hemoglobin" in label | | X5 : "mmol/l" in label | | X6 : "globin" in name | | X7 : "hb" in name | | X8 : "hemo" in name | | X9 : "hemoglobin" in name | | X10 : interquartile range < 29,59 | | X11 : interquartile range > 2,502 | | X12 : median < 10,4 | | X13 : median > 3,3 | | X14 : scale level is ratio | | ((((not X8) or (not X11)) or (X10 and X12)) and ((X6 and (not X7)) or (X12 and X4))) |
| Hba1c | | X1 : index(label,"%")>0 | | --- | | X2 : index(label,"1c")>0 | | X3 : index(label,"hba")>0 | | X4 : index(label,"hba")>0 & index(label,"1c") > 0 | | X5 : index(name,"%")>0 | | X6 : index(name,"1c")>0 | | X7 : index(name,"hba")>0 | | X8 : index(name,"hba")>0 & index(name,"1c") > 0 | | X9 : var_iqr < 1,3 | | X10 : var_iqr > 0,3999 | | X11 : var_med < 7,7787 | | X12 : var_med > 4,4444 | | X13 : (var_s = 1) > 0 | | | X1 : "%" in label | | --- | | X2 : "1c" in label | | X3 : "hba" in label | | X4 : "hba" in label and "1c" in label | | X5 : "%" in name | | X6 : "1c" in name | | X7 : "hba" in name | | X8 : "hba" in name and "1c" in name | | X9 : interquartile range < 1,3 | | X10 : interquartile range > 0,3999 | | X11 : median < 7,7787 | | X12 : median > 4,4444 | | X13 : scale level is ratio | | ((X4 and (X9 and X10)) or (X8 and (X11 and (not X1)))) |
| HDL cholesterol | | X1 : index(label,"hdl")>0 | | --- | | X2 : index(label,"high")>0 & index(label,"density") > 0 | | X3 : index(label,"mg/dl")>0 | | X4 : index(name,"hdl")>0 | | X5 : index(name,"hdl")>0 & index(name,"chol") > 0 | | X6 : index(name,"high")>0 & index(name,"density") > 0 | | X7 : var_iqr < 70 | | X8 : var_iqr > 14,58 | | X9 : var_med < 63 | | X10 : var_med > 42 | | X11 : (var_s = 1) > 0 | | | X1 : "hdl" in label | | --- | | X2 : "high" in label and "density" in label | | X3 : "mg/dl" in label | | X4 : "hdl" in name | | X5 : "hdl" in name and "chol" in name | | X6 : "high" in name and "density" in name | | X7 : interquartile range < 70 | | X8 : interquartile range > 14,58 | | X9 : median < 63 | | X10 : median > 42 | | X11 : scale level is ratio | | (X1 and X10) |
| HDL cholesterol SI | | X1 : index(label,"hdl")>0 | | --- | | X2 : index(label,"hdl")>0 & index(label,"chol") > 0 | | X3 : index(label,"high")>0 & index(label,"density") > 0 | | X4 : index(label,"mmol/l")>0 | | X5 : index(name,"hdl")>0 | | X6 : index(name,"hdl")>0 & index(name,"chol") > 0 | | X7 : index(name,"high")>0 & index(name,"density") > 0 | | X8 : var_iqr < 0,625 | | X9 : var_iqr > 0,33 | | X10 : var_med < 1,49 | | X11 : var_med > 1,064 | | X12 : (var_s = 1) > 0 | | | X1 : "hdl" in label | | --- | | X2 : "hdl" in label and "chol" in label | | X3 : "high" in label and "density" in label | | X4 : "mmol/l" in label | | X5 : "hdl" in name | | X6 : "hdl" in name and "chol" in name | | X7 : "high" in name and "density" in name | | X8 : interquartile range < 0,625 | | X9 : interquartile range > 0,33 | | X10 : median < 1,49 | | X11 : median > 1,064 | | X12 : scale level is ratio | | (((X8 and X11) and (X10 and X9)) or ((X2 and X10) or (X5 and (not X2)))) |
| History of CVD | | X1 : index(label,"CHD")>0 | | --- | | X2 : index(label,"CVD")>0 | | X3 : index(label,"etiology")>0 | | X4 : index(label,"have you had")>0 & (index(label,"heart") > 0 | index(label,"stroke") >0) | | X5 : index(label,"hist")>0 | | X6 : index(label,"prev")>0 & index(label,"chd") > 0 | | X7 : index(label,"prev")>0 & index(label,"CVD") > 0 | | X8 : index(label,"prev")>0 & index(label,"mi") > 0 | | X9 : index(label,"prev")>0 & index(label,"strok") > 0 | | X10 : index(label,"previous")>0 | | X11 : index(name,"CHD")>0 | | X12 : index(name,"CVD")>0 | | X13 : index(name,"etiology")>0 | | X14 : index(name,"have you had")>0 & (index(name,"heart") > 0 | index(name,"stroke") >0) | | X15 : index(name,"hist")>0 | | X16 : index(name,"prev")>0 & index(name,"chd") > 0 | | X17 : index(name,"prev")>0 & index(name,"CVD") > 0 | | X18 : index(name,"prev")>0 & index(name,"mi") > 0 | | X19 : index(name,"prev")>0 & index(name,"strok") > 0 | | X20 : index(name,"previous")>0 | | X21 : var_nr < 2 | | X22 : var_nr > 2 | | X23 : var_pr < 99,4449 | | X24 : var_pr > 65,218 | | X25 : (var_s = 3) > 0 | | | X1 : "CHD" in label | | --- | | X2 : "CVD" in label | | X3 : "etiology" in label | | X4 : "have you had" in label and ("heart" in label or "stroke" in label) | | X5 : "hist" in label | | X6 : "prev" in label and "chd" in label | | X7 : "prev" in label and "CVD" in label | | X8 : "prev" in label and "mi" in label | | X9 : "prev" in label and "strok" in label | | X10 : "previous" in label | | X11 : "CHD" in name | | X12 : "CVD" in name | | X13 : "etiology" in name | | X14 : "have you had" in name and ("heart" in name or "stroke" in name) | | X15 : "hist" in name | | X16 : "prev" in name and "chd" in name | | X17 : "prev" in name and "CVD" in name | | X18 : "prev" in name and "mi" in name | | X19 : "prev" in name and "strok" in name | | X20 : "previous" in name | | X21 : number of values < 2 | | X22 : number of values > 2 | | X23 : proportion of most frequent caregory < 99,4449 | | X24 : proportion of most frequent caregory > 65,218 | | X25 : scale level is dichotomous nominal | | (((X5 and (not X16)) or X20) or (((not X21) and X23) and ((not X3) or X18))) |
| Hs-CRP | | X1 : index(label,"c-rea")>0 | | --- | | X2 : index(label,"crp")>0 | | X3 : index(label,"hscrp")>0 | | X4 : index(name,"c-rea")>0 | | X5 : index(name,"crp")>0 | | X6 : index(name,"hscrp")>0 | | X7 : var_iqr < 4,72 | | X8 : var_iqr > 1,43 | | X9 : var_med < 4,1 | | X10 : var_med > 0,11 | | X11 : (var_s = 1) > 0 | | | X1 : "c-rea" in label | | --- | | X2 : "crp" in label | | X3 : "hscrp" in label | | X4 : "c-rea" in name | | X5 : "crp" in name | | X6 : "hscrp" in name | | X7 : interquartile range < 4,72 | | X8 : interquartile range > 1,43 | | X9 : median < 4,1 | | X10 : median > 0,11 | | X11 : scale level is ratio | | (((X2 or X1) or X5) and X10) |
| Hypertension | | X1 : index(label,"erhöhter")>0 & index(label,"blutdruck")>0 | | --- | | X2 : index(label,"high blood")>0 | | X3 : index(label,"hochdruck")>0 | | X4 : index(label,"hyperten")>0 | | X5 : index(name,"ht")>0 | | X6 : index(name,"hyp")>0 | | X7 : index(name,"hyp")>0 | index(name,"ht")>0 | | X8 : index(name,"hypert")>0 | | X9 : var_nr < 3 | | X10 : var_nr > 1 | | X11 : var_pr < 82,6268 | | X12 : var_pr > 30,4186 | | X13 : (var_s = 3) > 0 | | | X1 : "erhöhter" in label and "blutdruck" in label | | --- | | X2 : "high blood" in label | | X3 : "hochdruck" in label | | X4 : "hyperten" in label | | X5 : "ht" in name | | X6 : "hyp" in name | | X7 : "hyp" in name or "ht" in name | | X8 : "hypert" in name | | X9 : number of values < 3 | | X10 : number of values > 1 | | X11 : proportion of most frequent caregory < 82,6268 | | X12 : proportion of most frequent caregory > 30,4186 | | X13 : scale level is dichotomous nominal | | (((X8 and X13) and ((not X3) or (not X7))) or ((X12 and X11) and (X10 or X1))) |
| Intima Media Thickness (IMT) | | X1 : index(label,"[cm]")>0 | | --- | | X2 : index(label,"acc")>0 | | X3 : index(label,"bif")>0 | | X4 : index(label,"bul")>0 | | X5 : index(label,"bulb")>0 | | X6 : index(label,"cca")>0 | | X7 : index(label,"ica")>0 | | X8 : index(label,"imt")>0 | | X9 : index(label,"imt")>0 & index(label,"max") > 0 | | X10 : index(label,"imt")>0 & index(label,"mean") > 0 | | X11 : index(label,"intima")>0 | | X12 : index(label,"left")>0 | | X13 : index(label,"link")>0 | | X14 : index(label,"max")>0 & index(label,"cc") > 0 | | X15 : index(label,"recht")>0 | | X16 : index(label,"right")>0 | | X17 : index(label,"thickness")>0 | | X18 : index(label,"wal")>0 & (index(label,"near") > 0 | index(label,"far")>0) | | X19 : index(name,"[mm]")>0 | | X20 : index(name,"acc")>0 | | X21 : index(name,"bif")>0 | | X22 : index(name,"bul")>0 | | X23 : index(name,"bulb")>0 | | X24 : index(name,"cca")>0 | | X25 : index(name,"ica")>0 | | X26 : index(name,"imt")>0 | | X27 : index(name,"imt")>0 & index(name,"max") > 0 | | X28 : index(name,"imt")>0 & index(name,"mean") > 0 | | X29 : index(name,"intima")>0 | | X30 : index(name,"left")>0 | | X31 : index(name,"link")>0 | | X32 : index(name,"max")>0 & index(name,"cc") > 0 | | X33 : index(name,"recht")>0 | | X34 : index(name,"right")>0 | | X35 : index(name,"thickness")>0 | | X36 : index(name,"wal")>0 & (index(name,"near") > 0 | index(name,"far")>0) | | X37 : var_iqr < 0,2299 | | X38 : var_iqr > 0 | | X39 : var_med < 0,13 | | X40 : var_med > -1,9278 | | X41 : (var_s = 1) > 0 | | | X1 : "[cm]" in label | | --- | | X2 : "acc" in label | | X3 : "bif" in label | | X4 : "bul" in label | | X5 : "bulb" in label | | X6 : "cca" in label | | X7 : "ica" in label | | X8 : "imt" in label | | X9 : "imt" in label and "max" in label | | X10 : "imt" in label and "mean" in label | | X11 : "intima" in label | | X12 : "left" in label | | X13 : "link" in label | | X14 : "max" in label and "cc" in label | | X15 : "recht" in label | | X16 : "right" in label | | X17 : "thickness" in label | | X18 : "wal" in label and ("near" in label  or "far" in label) | | X19 : "[mm]" in name | | X20 : "acc" in name | | X21 : "bif" in name | | X22 : "bul" in name | | X23 : "bulb" in name | | X24 : "cca" in name | | X25 : "ica" in name | | X26 : "imt" in name | | X27 : "imt" in name and "max" in name | | X28 : "imt" in name and "mean" in name | | X29 : "intima" in name | | X30 : "left" in name | | X31 : "link" in name | | X32 : "max" in name and "cc" in name | | X33 : "recht" in name | | X34 : "right" in name | | X35 : "thickness" in name | | X36 : "wal" in name and ("near" in name or "far" in name) | | X37 : interquartile range < 0,2299 | | X38 : interquartile range > 0 | | X39 : median < 0,13 | | X40 : median > -1,9278 | | X41 : scale level is ratio | | ((((not X30) and (not X34)) and (X39 and X40)) and (((not X41) and (not X13)) or (X38 and (not X25)))) |
| Intima Media Thickness (IMT) SI | | X1 : index(label,"[mm]")>0 | | --- | | X2 : index(label,"acc")>0 | | X3 : index(label,"bif")>0 | | X4 : index(label,"bul")>0 | | X5 : index(label,"bulb")>0 | | X6 : index(label,"cca")>0 | | X7 : index(label,"ica")>0 | | X8 : index(label,"imt")>0 | | X9 : index(label,"imt")>0 & index(label,"max") > 0 | | X10 : index(label,"imt")>0 & index(label,"mean") > 0 | | X11 : index(label,"intima")>0 | | X12 : index(label,"left")>0 | | X13 : index(label,"link")>0 | | X14 : index(label,"max")>0 & index(label,"cc") > 0 | | X15 : index(label,"recht")>0 | | X16 : index(label,"right")>0 | | X17 : index(label,"thickness")>0 | | X18 : index(label,"wal")>0 & (index(label,"near") > 0 |  index(label,"far")>0) | | X19 : index(name,"[mm]")>0 | | X20 : index(name,"acc")>0 | | X21 : index(name,"bif")>0 | | X22 : index(name,"bul")>0 | | X23 : index(name,"bulb")>0 | | X24 : index(name,"cca")>0 | | X25 : index(name,"ica")>0 | | X26 : index(name,"imt")>0 | | X27 : index(name,"imt")>0 & index(name,"max") > 0 | | X28 : index(name,"imt")>0 & index(name,"mean") > 0 | | X29 : index(name,"intima")>0 | | X30 : index(name,"left")>0 | | X31 : index(name,"link")>0 | | X32 : index(name,"max")>0 & index(name,"cc") > 0 | | X33 : index(name,"recht")>0 | | X34 : index(name,"right")>0 | | X35 : index(name,"thickness")>0 | | X36 : index(name,"wal")>0 & (index(name,"near") > 0 | index(name,"far")>0) | | X37 : var_iqr < 1,4299 | | X38 : var_iqr > 0,1 | | X39 : var_med < 1,76 | | X40 : var_med > 0,0001 | | X41 : (var_s = 1) > 0 | | | X1 : "[mm]" in label | | --- | | X2 : "acc" in label | | X3 : "bif" in label | | X4 : "bul" in label | | X5 : "bulb" in label | | X6 : "cca" in label | | X7 : "ica" in label | | X8 : "imt" in label | | X9 : "imt" in label and "max" in label | | X10 : "imt" in label and "mean" in label | | X11 : "intima" in label | | X12 : "left" in label | | X13 : "link" in label | | X14 : "max" in label and "cc" in label | | X15 : "recht" in label | | X16 : "right" in label | | X17 : "thickness" in label | | X18 : "wal" in label and ("near" in label  or "far" in label) | | X19 : "[mm]" in name | | X20 : "acc" in name | | X21 : "bif" in name | | X22 : "bul" in name | | X23 : "bulb" in name | | X24 : "cca" in name | | X25 : "ica" in name | | X26 : "imt" in name | | X27 : "imt" in name and "max" in name | | X28 : "imt" in name and "mean" in name | | X29 : "intima" in name | | X30 : "left" in name | | X31 : "link" in name | | X32 : "max" in name and "cc" in name | | X33 : "recht" in name | | X34 : "right" in name | | X35 : "thickness" in name | | X36 : "wal" in name and ("near" in name  or "far" in name) | | X37 : interquartile range < 1,4299 | | X38 : interquartile range > 0,1 | | X39 : median < 1,76 | | X40 : median > 0,0001 | | X41 : scale level is ratio | | (((X40 and X39) and (X32 or X38)) or ((X26 and (not X37)) and (not X6))) |
| Arterial diameter | | X1 : index(label,"dia")>0 & index(label,"cc") > 0 | | --- | | X2 : index(label,"dia")>0 & index(label,"cca") > 0 | | X3 : index(label,"dia")>0 & index(label,"ica") > 0 | | X4 : index(label,"diam")>0 | | X5 : index(label,"diameter")>0 | | X6 : index(label,"lumen")>0 | | X7 : index(name,"dia")>0 & index(name,"cc") > 0 | | X8 : index(name,"dia")>0 & index(name,"cca") > 0 | | X9 : index(name,"dia")>0 & index(name,"ica") > 0 | | X10 : index(name,"diam")>0 | | X11 : index(name,"diameter")>0 | | X12 : index(name,"lumen")>0 | | X13 : var_iqr < 3 | | X14 : var_iqr > 0,75 | | X15 : var_med < 12,8 | | X16 : var_med > 4,71 | | X17 : (var_s = 1) > 0 | | | X1 : "dia" in label and "cc" in label | | --- | | X2 : "dia" in label and "cca" in label | | X3 : "dia" in label and "ica" in label | | X4 : "diam" in label | | X5 : "diameter" in label | | X6 : "lumen" in label | | X7 : "dia" in name and "cc" in name | | X8 : "dia" in name and "cca" in name | | X9 : "dia" in name and "ica" in name | | X10 : "diam" in name | | X11 : "diameter" in name | | X12 : "lumen" in name | | X13 : interquartile range < 3 | | X14 : interquartile range > 0,75 | | X15 : median < 12,8 | | X16 : median > 4,71 | | X17 : scale level is ratio | | (((X13 and X14) and (X16 and X15)) or X5) |
| Income | | X1 : index(label,"einko")>0 | | --- | | X2 : index(label,"inco")>0 | | X3 : index(label,"verd")>0 | | X4 : index(name,"einko")>0 | | X5 : index(name,"inco")>0 | | X6 : index(name,"verd")>0 | | X7 : var_nr < 8 | | X8 : var_nr > 1 | | X9 : var_pr < 72,2892 | | X10 : var_pr > 18,1727 | | X11 : (var_s = 2) > 0 | | | X1 : "einko" in label | | --- | | X2 : "inco" in label | | X3 : "verd" in label | | X4 : "einko" in name | | X5 : "inco" in name | | X6 : "verd" in name | | X7 : number of values < 8 | | X8 : number of values > 1 | | X9 : proportion of most frequent caregory < 72,2892 | | X10 : proportion of most frequent caregory > 18,1727 | | X11 : scale level is ordinal or nominal | | (X2 and X10) |
| LDL cholesterol | | X1 : index(label,"ldl")>0 | | --- | | X2 : index(label,"ldl")>0 & index(label,"chol") > 0 | | X3 : index(label,"low")>0 & index(label,"density") > 0 | | X4 : index(label,"mg/dl")>0 | | X5 : index(name,"ldl")>0 | | X6 : index(name,"ldl")>0 & index(name,"chol") > 0 | | X7 : index(name,"low")>0 & index(name,"density") > 0 | | X8 : var_iqr < 151 | | X9 : var_iqr > 38 | | X10 : var_med < 159 | | X11 : var_med > 108 | | X12 : (var_s = 1) > 0 | | | X1 : "ldl" in label | | --- | | X2 : "ldl" in label and "chol" in label | | X3 : "low" in label and "density" in label | | X4 : "mg/dl" in label | | X5 : "ldl" in name | | X6 : "ldl" in name and "chol" in name | | X7 : "low" in name and "density" in name | | X8 : interquartile range < 151 | | X9 : interquartile range > 38 | | X10 : median < 159 | | X11 : median > 108 | | X12 : scale level is ratio | | (X5 and X11) |
| LDL cholesterol SI | | X1 : index(label,"ldl")>0 | | --- | | X2 : index(label,"ldl")>0 & index(label,"chol") > 0 | | X3 : index(label,"low")>0 & index(label,"density") > 0 | | X4 : index(label,"mmol/l")>0 | | X5 : index(name,"ldl")>0 | | X6 : index(name,"ldl")>0 & index(name,"chol") > 0 | | X7 : index(name,"low")>0 & index(name,"density") > 0 | | X8 : var_iqr < 1,9 | | X9 : var_iqr > 0,9709 | | X10 : var_med < 5,21 | | X11 : var_med > 2,2105 | | X12 : (var_s = 1) > 0 | | | X1 : "ldl" in label | | --- | | X2 : "ldl" in label and "chol" in label | | X3 : "low" in label and "density" in label | | X4 : "mmol/l" in label | | X5 : "ldl" in name | | X6 : "ldl" in name and "chol" in name | | X7 : "low" in name and "density" in name | | X8 : interquartile range < 1,9 | | X9 : interquartile range > 0,9709 | | X10 : median < 5,21 | | X11 : median > 2,2105 | | X12 : scale level is ratio | | (((X1 and (not X2)) or (X2 and (not X9))) or ((X8 and X9) and (X10 and X11))) |
| Leukocytes | | X1 : index(label,"leu")>0 | | --- | | X2 : index(label,"leuco")>0 | | X3 : index(label,"leuko")>0 | | X4 : index(label,"wbc")>0 | | X5 : index(label,"white")>0 & index(label,"blood") > 0 | | X6 : index(name,"leu")>0 | | X7 : index(name,"leuco")>0 | | X8 : index(name,"leuko")>0 | | X9 : index(name,"wbc")>0 | | X10 : index(name,"white")>0 & index(name,"blood") > 0 | | X11 : var_iqr < 2,4 | | X12 : var_iqr > 1,61 | | X13 : var_med < 6,4 | | X14 : var_med > 5,1399 | | X15 : (var_s = 1) > 0 | | | X1 : "leu" in label | | --- | | X2 : "leuco" in label | | X3 : "leuko" in label | | X4 : "wbc" in label | | X5 : "white" in label and "blood" in label | | X6 : "leu" in name | | X7 : "leuco" in name | | X8 : "leuko" in name | | X9 : "wbc" in name | | X10 : "white" in name and "blood" in name | | X11 : interquartile range < 2,4 | | X12 : interquartile range > 1,61 | | X13 : median < 6,4 | | X14 : median > 5,1399 | | X15 : scale level is ratio | | ((X6 or X1) or (X13 and X5)) |
| Dyslipidemia | | X1 : index(label,"dyslip")>0 | | --- | | X2 : index(label,"hyperchol")>0 & index(label,"med") = 0 | | X3 : index(label,"lip")>0 & index(label,"abn") > 0 | | X4 : index(label,"lipid")>0 & index(label,"med") = 0 | | X5 : index(name,"dyslip")>0 | | X6 : index(name,"hyperchol")>0 & index(name,"med") = 0 | | X7 : index(name,"lip")>0 & index(name,"abn") > 0 | | X8 : index(name,"lipid")>0 & index(name,"med") = 0 | | X9 : var_nr < 5 | | X10 : var_nr > 1 | | X11 : var_pr < 63,0045 | | X12 : var_pr > 19,1662 | | X13 : (var_s = 3) > 0 | | | X1 : "dyslip" in label | | --- | | X2 : "hyperchol" in label and not "med" in label | | X3 : "lip" in label and "abn" in label | | X4 : "lipid" in label and not "med" in label | | X5 : "dyslip" in name | | X6 : "hyperchol" in name and not "med" in name | | X7 : "lip" in name and "abn" in name | | X8 : "lipid" in name and not "med" in name | | X9 : number of values < 5 | | X10 : number of values > 1 | | X11 : proportion of most frequent caregory < 63,0045 | | X12 : proportion of most frequent caregory > 19,1662 | | X13 : scale level is dichotomous nominal | | (((X3 or X5) and (X11 or (not X3))) or ((X13 and X10) and (X12 and X11))) |
| Antidiabetic medication | | X1 : index(label,"agl")>0 | | --- | | X2 : index(label,"antidia")>0 | | X3 : index(label,"antidia")>0 | | X4 : index(label,"dia")>0 & index(label,"bahandlung") > 0 | | X5 : index(label,"dia")>0 & index(label,"drug") > 0 | | X6 : index(label,"dia")>0 & index(label,"med") > 0 | | X7 : index(label,"dia")>0 & index(label,"tablet") > 0 | | X8 : index(label,"gluc")>0 & index(label,"low") > 0 | | X9 : index(label,"hypoglyc")>0 | | X10 : index(label,"insul")>0 | | X11 : index(label,"piogli")>0 | | X12 : index(label,"therdia")>0 | | X13 : index(name,"agl")>0 | | X14 : index(name,"antidia")>0 | | X15 : index(name,"antidia")>0 | | X16 : index(name,"dia")>0 & index(name,"bahandlung") > 0 | | X17 : index(name,"dia")>0 & index(name,"drug") > 0 | | X18 : index(name,"dia")>0 & index(name,"med") > 0 | | X19 : index(name,"dia")>0 & index(name,"tablet") > 0 | | X20 : index(name,"gluc")>0 & index(name,"low") > 0 | | X21 : index(name,"hypoglyc")>0 | | X22 : index(name,"insul")>0 | | X23 : index(name,"piogli")>0 | | X24 : index(name,"therdia")>0 | | X25 : var_nr < 3 | | X26 : var_nr > 5 | | X27 : var_pr < 99,6564 | | X28 : var_pr > 40,0286 | | X29 : (var_s = 2) > 0 | | | X1 : "agl" in label | | --- | | X2 : "antidia" in label | | X3 : "antidia" in label | | X4 : "dia" in label and "behandlung" in label | | X5 : "dia" in label and "drug" in label | | X6 : "dia" in label and "med" in label | | X7 : "dia" in label and "tablet" in label | | X8 : "gluc" in label and "low" in label | | X9 : "hypoglyc" in label | | X10 : "insul" in label | | X11 : "piogli" in label | | X12 : "therdia" in label | | X13 : "agl" in name | | X14 : "antidi" in name | | X15 : "antidia" in name | | X16 : "dia" in name and "behandlung" in name | | X17 : "dia" in name and "drug" in name | | X18 : "dia" in name and "med" in name | | X19 : "dia" in name and "tablet" in name | | X20 : "gluc" in name and "low" in name | | X21 : "hypoglyc" in name | | X22 : "insul" in name | | X23 : "piogli" in name | | X24 : "therdia" in name | | X25 : number of values < 3 | | X26 : number of values > 5 | | X27 : proportion of most frequent caregory < 99,6564 | | X28 : proportion of most frequent caregory > 40,0286 | | X29 : scale level is ordinal or nominal | | (((X22 or X2) and ((not X4) or (not X14))) or ((X13 and (not X1)) or ((not X26) and X27))) |
| Antihypertensive medication | | X1 : index(label,"ace")>0 | | --- | | X2 : index(label,"angioten")>0 | | X3 : index(label,"antag")>0 | | X4 : index(label,"anti")>0 & index(label,"hyp")>0 | | X5 : index(label,"antihyp")>0 | | X6 : index(label,"beta")>0 | | X7 : index(label,"block")>0 | | X8 : index(label,"blokk")>0 | | X9 : index(label,"bp")>0 & index(label,"drug")>0 | | X10 : index(label,"diuretic")>0 | | X11 : index(label,"drug")>0 & index(label,"ht")>0 | | X12 : index(label,"ht")>0 & index(label,"med")>0 | | X13 : index(label,"hyp")>0 & index(label,"drug")>0 | | X14 : index(label,"med")>0 & index(label,"high blood")>0 | | X15 : index(label,"med")>0 & index(label,"ht")>0 | | X16 : index(label,"med")>0 & index(label,"hyp")>0 | | X17 : index(label,"mittel")>0 & index(label,"hyper")>0 | | X18 : index(label,"pressure")>0 & index(label,"low")>0 | | X19 : index(name,"ace")>0 | | X20 : index(name,"angioten")>0 | | X21 : index(name,"antag")>0 | | X22 : index(name,"anti")>0 & index(name,"hyp")>0 | | X23 : index(name,"antihyp")>0 | | X24 : index(name,"beta")>0 | | X25 : index(name,"block")>0 | | X26 : index(name,"blokk")>0 | | X27 : index(name,"bp")>0 & index(name,"drug")>0 | | X28 : index(name,"diuretic")>0 | | X29 : index(name,"drug")>0 & index(name,"ht")>0 | | X30 : index(name,"ht")>0 & index(name,"med")>0 | | X31 : index(name,"hyp")>0 & index(name,"drug")>0 | | X32 : index(name,"med")>0 & index(name,"high blood")>0 | | X33 : index(name,"med")>0 & index(name,"ht")>0 | | X34 : index(name,"med")>0 & index(name,"hyp")>0 | | X35 : index(name,"mittel")>0 & index(name,"hyper")>0 | | X36 : index(name,"pressure")>0 & index(name,"low")>0 | | X37 : var_nr < 2 | | X38 : var_nr > 4 | | X39 : var_pr < 94,1581 | | X40 : var_pr > 0,5737 | | X41 : (var_s = 2) > 0 | | | X1 : "ace" in label | | --- | | X2 : "angioten" in label | | X3 : "antag" in label | | X4 : "anti" in label and "hyp" in label | | X5 : "antihyp" in label | | X6 : "beta" in label | | X7 : "block" in label | | X8 : "blokk" in label | | X9 : "bp" in label and "drug" in label | | X10 : "diuretic" in label | | X11 : "drug" in label and "ht" in label | | X12 : "ht" in label and "med" in label | | X13 : "hyp" in label and "drug" in label | | X14 : "med" in label and "high blood" in label | | X15 : "med" in label and "ht" in label | | X16 : "med" in label and "hyp" in label | | X17 : "mittel" in label and "hyper" in label | | X18 : "pressure" in label and "low" in label | | X19 : "ace" in name | | X20 : "angioten" in name | | X21 : "antag" in name | | X22 : "anti" in name and "hyp" in name | | X23 : "antihyp" in name | | X24 : "beta" in name | | X25 : "block" in name | | X26 : "blokk" in name | | X27 : "bp" in name and "drug" in name | | X28 : "diuretic" in name | | X29 : "drug" in name and "ht" in name | | X30 : "ht" in name and "med" in name | | X31 : "hyp" in name and "drug" in name | | X32 : "med" in name and "high blood" in name | | X33 : "med" in name and "ht" in name | | X34 : "med" in name and "hyp" in name | | X35 : "mittel" in name and "hyper" in name | | X36 : "pressure" in name and "low" in name | | X37 : number of values < 2 | | X38 : number of values > 4 | | X39 : proportion of most frequent caregory < 94,1581 | | X40 : proportion of most frequent caregory > 0,5737 | | X41 : scale level is ordinal or nominal | | (((X40 and (not X15)) and (X39 and (not X37))) or ((X4 or X3) or X33)) |
| Lipid-lowering medication | | X1 : index(label,"astin")>0 | | --- | | X2 : index(label,"drug")>0 & index(label,"lip") > 0 | | X3 : index(label,"fibrat")>0 | | X4 : index(label,"lip")>0 & (index(label,"med") > 0 | index(label,"low")>0) | | X5 : index(label,"lip")>0 & index(label,"low") > 0 & index(label,"med")>0 | | X6 : index(label,"lipid")>0 | | X7 : index(label,"low")>0 & index(label,"lip") > 0 | | X8 : index(label,"med")>0 & index(label,"chol") > 0 | | X9 : index(label,"med")>0 & index(label,"lip") > 0 | | X10 : index(label,"resin")>0 | | X11 : index(label,"stati")>0 | | X12 : index(name,"astin")>0 | | X13 : index(name,"drug")>0 & index(name,"lip") > 0 | | X14 : index(name,"fibrat")>0 | | X15 : index(name,"lip")>0 & (index(name,"med") > 0 | index(name,"low")>0) | | X16 : index(name,"lip")>0 & index(name,"low") > 0 & index(name,"med")>0 | | X17 : index(name,"lipid")>0 | | X18 : index(name,"low")>0 & index(name,"lip") > 0 | | X19 : index(name,"med")>0 & index(name,"chol") > 0 | | X20 : index(name,"med")>0 & index(name,"lip") > 0 | | X21 : index(name,"resin")>0 | | X22 : index(name,"stati")>0 | | X23 : var_nr < 2 | | X24 : var_nr > 3 | | X25 : var_pr < 99,8975 | | X26 : var_pr > 0,0628 | | X27 : (var_s = 2) > 0 | | | X1 : "astin" in label | | --- | | X2 : "drug" in label and "lip" in label | | X3 : "fibrat" in label | | X4 : "lip" in label and ("med" in label or "low" in label) | | X5 : "lip" in label and "low" in label and "med" in label | | X6 : "lipid" in label | | X7 : "low" in label and "lip" in label | | X8 : "med" in label and "chol" in label | | X9 : "med" in label and "lip" in label | | X10 : "resin" in label | | X11 : "stati" in label | | X12 : "astin" in name | | X13 : "drug" in name and "lip" in name | | X14 : "fibrat" in name | | X15 : "lip" in name and ("med" in name  or "low" in name) | | X16 : "lip" in name and "low" in name  and "med" in name | | X17 : "lipid" in name | | X18 : "low" in name and "lip" in name | | X19 : "med" in name and "chol" in name | | X20 : "med" in name and "lip" in name | | X21 : "resin" in name | | X22 : "stati" in name | | X23 : number of values < 2 | | X24 : number of values > 3 | | X25 : proportion of most frequent caregory < 99,8975 | | X26 : proportion of most frequent caregory > 0,0628 | | X27 : scale level is ordinal or nominal | | ((((not X25) and (not X6)) or X4) or (((not X23) and X26) and ((not X27) and (not X17)))) |
| Nicotine consumption | | X1 : index(label,"pack")>0 & index(label,"year") > 0 | | --- | | X2 : index(label,"packy")>0 | | X3 : index(label,"pcky")>0 | | X4 : index(label,"smoke")>0 & index(label,"year") > 0 | | X5 : index(name,"pack")>0 & index(name,"year") > 0 | | X6 : index(name,"packy")>0 | | X7 : index(name,"pcky")>0 | | X8 : index(name,"smoke")>0 & index(name,"year") > 0 | | X9 : var_iqr < 7208,75 | | X10 : var_iqr > 5,6465 | | X11 : var_med < 7710,625 | | X12 : var_med > -0,0137 | | X13 : (var_s = 1) > 0 | | | X1 : "pack" in label and "year" in label | | --- | | X2 : "packy" in label | | X3 : "pcky" in label | | X4 : "smoke" in label and "year" in label | | X5 : "pack" in name and "year" in name | | X6 : "packy" in name | | X7 : "pcky" in name | | X8 : "smoke" in name and "year" in name | | X9 : interquartile range < 7208,75 | | X10 : interquartile range > 5,6465 | | X11 : median < 7710,625 | | X12 : median > -0,0137 | | X13 : scale level is ratio | | (((X12 and (not X8)) and (X11 and X10)) or ((X6 or X1) or (X8 and (not X4)))) |
| Carotid plaque | | X1 : index(label,"plaq")>0 | | --- | | X2 : index(label,"plaq")>0 & index(label,"adjust") = 0 | | X3 : index(name,"plaq")>0 | | X4 : index(name,"plaq")>0 & index(name,"adjust") = 0 | | X5 : var_nr < 4 | | X6 : var_nr > 1 | | X7 : var_pr < 97,2054 | | X8 : var_pr > 24,0223 | | X9 : (var_s = 1) > 0 | | | X1 : "plaq" in label | | --- | | X2 : "plaq" in label and not "adjust" in label | | X3 : "plaq" in name | | X4 : "plaq" in name and not "adjust" in name | | X5 : number of values < 4 | | X6 : number of values > 1 | | X7 : proportion of most frequent caregory < 97,2054 | | X8 : proportion of most frequent caregory > 24,0223 | | X9 : scale level is ratio | | (((X5 or X1) and (X8 and X7)) or ((X3 or X9) or ((not X8) and (not X5)))) |
| Diastolic blood pressure | | X1 : index(label,"bddia")>0 | | --- | | X2 : index(label,"db")>0 | | X3 : index(label,"dbp")>0 | | X4 : index(label,"dia")>0 | | X5 : index(label,"diabd")>0 | | X6 : index(label,"diast")>0 | | X7 : index(name,"bddia")>0 | | X8 : index(name,"db")>0 | | X9 : index(name,"dbp")>0 | | X10 : index(name,"dia")>0 | | X11 : index(name,"diabd")>0 | | X12 : index(name,"diast")>0 | | X13 : var_iqr < 20 | | X14 : var_iqr > 9,9033 | | X15 : var_med < 90 | | X16 : var_med > 64,4408 | | X17 : (var_s = 1) > 0 | | | X1 : "bddia" in label | | --- | | X2 : "db" in label | | X3 : "dbp" in label | | X4 : "dia" in label | | X5 : "diabd" in label | | X6 : "diast" in label | | X7 : "bddia" in label | | X8 : "db" in name | | X9 : "dbp" in name | | X10 : "dia" in name | | X11 : "diabd" in name | | X12 : "diast" in name | | X13 : interquartile range < 20 | | X14 : interquartile range > 9,9033 | | X15 : median < 90 | | X16 : median > 64,4408 | | X17 : scale level is ratio | | (((X8 or X1) and (X16 and X15)) or X6) |
| Systolic blood pressure | | X1 : index(label,"bdsys")>0 | | --- | | X2 : index(label,"sb")>0 | | X3 : index(label,"sbp")>0 | | X4 : index(label,"sys")>0 | | X5 : index(label,"sysbd")>0 | | X6 : index(label,"syst")>0 | | X7 : index(name,"bdsys")>0 | | X8 : index(name,"sb")>0 | | X9 : index(name,"sbp")>0 | | X10 : index(name,"sys")>0 | | X11 : index(name,"sysbd")>0 | | X12 : index(name,"syst")>0 | | X13 : var_iqr < 856 | | X14 : var_iqr > 14,5 | | X15 : var_med < 166 | | X16 : var_med > 112,4632 | | X17 : (var_s = 1) > 0 | | | X1 : "bdsys" in label | | --- | | X2 : "sb" in label | | X3 : "sbp" in label | | X4 : "sys" in label | | X5 : "sysbd" in label | | X6 : "syst" in label | | X7 : "bdsys" in name | | X8 : "sb" in name | | X9 : "sbp" in name | | X10 : "sys" in name | | X11 : "sysbd" in name | | X12 : "syst" in name | | X13 : interquartile range < 856 | | X14 : interquartile range > 14,5 | | X15 : median < 166 | | X16 : median > 112,4632 | | X17 : scale level is ratio | | (((X8 or X6) and (X14 and (not X6))) or ((X10 or X9) and (X6 or X7))) |
| Socioeconomic status | | X1 : var_nr < 7 | | --- | | X2 : var_nr > 1 | | X3 : var_pr < 86,6231 | | X4 : (var_s = 2) > 0 | | | X1 : number of values < 7 | | --- | | X2 : number of values > 1 | | X3 : proportion of most frequent caregory < 86,6231 | | X4 : scale level is ordinal or nominal | | ((X4 or (not X2)) and X3) |
| Sex | | X5 : index(label,"gend")>0 | | --- | | X1 : index(label,"geschlecht")>0 | | X2 : index(label,"sex")>0 | | X3 : index(name,"gend")>0 | | X4 : index(name,"geschlecht")>0 | | X5 : index(name,"sex")>0 | | X6 : index(name,"sex")>0 | index(name,"gend")>0 | | X7 : var_nr < 2 | | X8 : var_nr > 1 | | X9 : var_pr < 60,481 | | X10 : var_pr > 3,9229 | | X11 : (var_s = 3) > 0 | | | X5 : "gend" in label | | --- | | X1 : "geschlecht" in label | | X2 : "sex" in label | | X3 : "gend" in name | | X4 : "geschlecht" in name | | X5 : "sex" in name | | X6 : "sex" in name or "gend" in name | | X7 : number of values < 2 | | X8 : number of values > 1 | | X9 : proportion of most frequent caregory < 60,481 | | X10 : proportion of most frequent caregory > 3,9229 | | X11 : scale level is dichotomous nominal | | X7 |
| Smoking status | | X1 : index(label,"cig")>0 | | --- | | X2 : index(label,"nic")>0 | | X3 : index(label,"nik")>0 | | X4 : index(label,"pipe")>0 | | X5 : index(label,"rauch")>0 | | X6 : index(label,"smok")>0 | | X7 : index(label,"zig")>0 | | X8 : index(name,"cig")>0 | | X9 : index(name,"nic")>0 | | X10 : index(name,"nik")>0 | | X11 : index(name,"pipe")>0 | | X12 : index(name,"rauch")>0 | | X13 : index(name,"smok")>0 | | X14 : index(name,"zig")>0 | | X15 : var_nr < 4 | | X16 : var_nr > 1 | | X17 : var_pr < 80,0051 | | X18 : var_pr > 5,1538 | | X19 : (var_s = 2) > 0 | | | X1 : "cig" in label | | --- | | X2 : "nic" in label | | X3 : "nik" in label | | X4 : "pipe" in label | | X5 : "rauch" in label | | X6 : "smok" in label | | X7 : "zig" in label | | X8 : "cig" in name | | X9 : "nic" in name | | X10 : "nik" in name | | X11 : "pipe" in name | | X12 : "rauch" in name | | X13 : "smok" in name | | X14 : "zig" in name | | X15 : number of values < 4 | | X16 : number of values > 1 | | X17 : proportion of most frequent caregory < 80,0051 | | X18 : proportion of most frequent caregory > 5,1538 | | X19 : scale level is ordinal or nominal | | (((X18 or X14) or (X9 and X5)) and ((X17 and (not X4)) or ((not X19) and X16))) |
| Triglycerides | | X1 : index(label,"gly")>0 | | --- | | X2 : index(label,"glycer")>0 | | X3 : index(label,"tg")>0 | | X4 : index(label,"trg")>0 | | X5 : index(label,"trig")>0 | | X6 : index(label,"trigl")>0 | | X7 : index(name,"gly")>0 | | X8 : index(name,"glycer")>0 | | X9 : index(name,"tg")>0 | | X10 : index(name,"trg")>0 | | X11 : index(name,"trig")>0 | | X12 : index(name,"trigl")>0 | | X13 : var_iqr < 109 | | X14 : var_iqr > 0,65 | | X15 : var_med < 151 | | X16 : var_med > 1,064 | | X17 : (var_s = 1) > 0 | | | X1 : "gly" in label | | --- | | X2 : "glycer" in label | | X3 : "tg" in label | | X4 : "trg" in label | | X5 : "trig" in label | | X6 : "trigl" in label | | X7 : "gly" in name | | X8 : "glycer" in name | | X9 : "tg" in name | | X10 : "trg" in name | | X11 : "trig" in name | | X12 : "trigl" in name | | X13 : interquartile range < 109 | | X14 : interquartile range > 0,65 | | X15 : median < 151 | | X16 : median > 1,064 | | X17 : scale level is ratio | | (((X14 and X16) and (X11 or X13)) and ((X15 and (not X7)) or (X5 or X4))) |
| Ultrasound date | | X1 : index(label,"dat")>0 & index(label,"onderzoek") > 0 | | --- | | X2 : index(label,"date")>0 | | X3 : index(label,"date")>0 & index(label,"exam") > 0 | | X4 : index(label,"date")>0 & index(label,"imt") > 0 | | X5 : index(label,"date")>0 & index(label,"ultra") > 0 | | X6 : index(label,"date")>0 & index(label,"us") > 0 | | X7 : index(label,"date")>0 & index(label,"visit") > 0 | | X8 : index(label,"datum")>0 & index(label,"unter") > 0 | | X9 : index(label,"zeit")>0 & index(label,"unter") > 0 | | X10 : index(name,"dat")>0 & index(name,"onderzoek") > 0 | | X11 : index(name,"date")>0 | | X12 : index(name,"date")>0 & index(name,"exam") > 0 | | X13 : index(name,"date")>0 & index(name,"imt") > 0 | | X14 : index(name,"date")>0 & index(name,"ultra") > 0 | | X15 : index(name,"date")>0 & index(name,"us") > 0 | | X16 : index(name,"date")>0 & index(name,"visit") > 0 | | X17 : index(name,"datum")>0 & index(name,"unter") > 0 | | X18 : index(name,"zeit")>0 & index(name,"unter") > 0 | | X19 : var_iqr < 19389 | | X20 : var_iqr > 17,5 | | X21 : var_med < 20071107,5 | | X22 : var_med > 10258 | | X23 : (var_s = 1) > 0 | | | X1 : "dat" in label and "onderzoek" in label | | --- | | X2 : "date" in label | | X3 : "date" in label and "exam" in label | | X4 : "date" in label and "imt" in label | | X5 : "date" in label and "ultra" in label | | X6 : "date" in label and "us" in label | | X7 : "date" in label and "visit" in label | | X8 : "datum" in label and "unter" in label | | X9 : "zeit" in label and "unter" in label | | X10 : "dat" in name and "onderzoek" in name | | X11 : "date" in name | | X12 : "date" in name and "exam" in name | | X13 : "date" in name and "imt" in name | | X14 : "date" in name and "ultra" in name | | X15 : "date" in name and "us" in name | | X16 : "date" in name and "visit" in name | | X17 : "datum" in name and "unter" in name | | X18 : "zeit" in name and "unter" in name | | X19 : interquartile range < 19389 | | X20 : interquartile range > 17,5 | | X21 : median < 20071107,5 | | X22 : median > 10258 | | X23 : scale level is ratio | | ((((not X11) or X5) and X19) or (((not X12) and X2) and (not X14))) |

Table S2: Program parameters

| **Parameter** | **Value** | **Implication** |
| --- | --- | --- |
| type | 1|3 | Classification | Logistic regression |
| select | 1 | Single model |
| n trees | 2 | Number of logic trees to be fit |
| scoring function | default | Algorithm minimizes false positive allocation |
| start | -1 | Upper temperature |
| end | -4 | Lower temperature |
| iter | 25000 | Total number of iterations in the annealing chain |
| update | 1000 | Number of iterations after which the scores are updated |

**List of Members of the PROG-IMT Study group**

**Status as of 15^th^ december 2015**

Stefan Agewall, MD, PhD, Prof.

Institute of Clinical Sciences,University of Oslo, Oslo, Norway

and

Department of Cardiology, Oslo University Hospital Ullevål, Oslo, Norway

Tadao Akizawa, MD, PhD, Prof

Division of Nephrology,Department of Medicine,Showa University School of Medicine, Tokyo, Japan

Mayuko Amaha, MD

Division of Nephrology,Department of Internal Medicine,Shinmatsudo Central General Hospital, Chiba, Japan

Mauro Amato, PhD

Centro Cardiologico Monzino, IRCCS, Milan, Italy

Steen Andersen, MD, DMSc

Steno Diabetes Center, Gentofte, Denmark

Sigmund A. Anderssen, PhD, Prof

Norwegian School of Sports Sciences, Oslo, Norway

Aleksandra Araszkiewicz, MD, PhD, Assoc. Prof.

Department of Internal Medicine and Diabetology, Poznan University of Medical Sciences, Poznan, Poland

Folkert W. Asselbergs, MD, PhD, Assoc. Prof.

Department of Cardiology,Division Heart & Lungs,University Medical Center Utrecht, Utrecht, the Netherlands

Gülay Asçi, Prof. Dr.

Nefroloji Bilim Dali,Tip Fakültesi,Ege Üniversitesi, Bornova-Izmir, Turkey

Jang-Ho Bae, MD.,PhD.,FACC.

College of Medicine, Konyang University Hospital, Daejeon, Korea

and

Heart Center, Konyang University Hospital, Daejeon, Korea

Tatyana Balakhonova, MD, PhD, Prof.

Ultrasound Vascular Laboratory,Cardiology Research Center , Moscow, Russia

Damiano Baldassarre, PhD, Prof

Centro Cardiologico Monzino, IRCCS, Milan, Italy

and

Dipartimento di Scienze Farmacologiche e Biomolecolari, Università di Milano, Milan, Italy

Edith Beishuizen

Department of General Internal Medicine, Leiden University Medical Center, Leiden, the Netherlands

Oscar Beloqui, MD, PhD

Department of Internal Medicine, University Clinic of Navarra, Navarra, Spain

Gerald Berenson, MD, Prof.

Department of Medicine, Pediatrics, Biochemistry, Epidemiology, Tulane University School of Medicine and School of Public Health and Tropical Medicine, New Orleans, USA

Göran Bergström, MD, PhD, Prof

Wallenberg Laboratory for Cardiovascular Research, Sahlgrenska Academy, Gothenburg University, Götheborg, Sweden

Enrique Bernal, MD, PhD

Infectious Diseases Unit,Hospital Reina Sofía , Murcia, Spain

Sebastjan Bevc, MD, PhD, Assist Prof

Department of Nephrology,Clinic for Internal Medicine,University Medical Centre Maribor, Maribor, Slovenia

Lokpal Bhatia, MB BCh

Faculty of Medicine,Human Development & Health Academic Unit,University of Southampton - Southampton General Hospital, Southhampton, UK

and

Southampton NIHR Biomedical Research Centre, University Hospital Southampton - Southampton General Hospital, ,

Horst Bickel, PhD

Department of Psychiatry and Psychotherapy, Technische Universität München, Munich, Germany

Stefan Blankenberg, MD, Prof.

2nd Department of Medicine, Johannes Gutenberg-Universität, Mainz, Germany

and

Department of Cardiology, University Hospital Hamburg-Eppendorf, Hamburg, Germany

Peter J. Blankestijn

Department of Nephrology, University Medical Center Utrecht, Utrecht, The Netherlands

James A Blumenthal, PhD

Department of Psychiatry and Behavioral Sciences, Duke University Medical Center, Durham, NC 27710, USA

Lena Bokemark, MD, PhD

Wallenberg Laboratory for Cardiovascular Research,Institution for Medicin, Department for Molecular and Clinical Medicine,Sahlgrenska Academy, Gothenburg University, Gothenburg, Sweden

Jackie Bosch, MSc

Population Health Research Institute, McMaster University, Hamilton, Ontario, Canada

Michiel Bots, MD, PhD, Prof

Julius Center for Health Sciences and Primary Care, University Medical Center Utrecht, Utrecht, the Netherlands

Frank P. Brouwers, MD, PhD

Department of Cardiology, University Medical Center Groningen, Groningen, the Netherlands

Christopher D. Byrne, MB BCh, PhD, Prof.

Faculty of Medicine,Human Development & Health Academic Unit,University of Southampton - Southampton General Hospital, Southhampton, UK

and

Southampton NIHR Biomedical Research Centre, University Hospital Southampton - Southampton General Hospital, ,

Alpaslan Bülbül, MPH

Department of Neurology, Goethe University, Frankfurt am Main, Germany

Philip C. Calder, PhD, Prof.

Faculty of Medicine,Human Development & Health Academic Unit,University of Southampton - Southampton General Hospital, Southhampton, UK

and

Southampton NIHR Biomedical Research Centre, University Hospital Southampton - Southampton General Hospital, ,

Yong-Jun Cao, MD, PhD

Department of Neurology,Institute of Neuroscience,the Second Affiliated Hospital of Soochow University, Soochow, China

Samuela Castelnuovo, PhD

Centro Dislipidemie E. Grossi Paoletti,Ospedale Ca’ Granda di Niguarda , Milan, Italy

Alberico Catapano, PhD, Prof.

IRCSS Multimedica, Milan, Italy

and

Department of Pharmacological and Biomolecular Sciences, University of Milan, Milan, Italy

Chen-Huan Chen, Prof, MD

National Yang-Ming University, Taipei, Taiwan

Kuo-Liong Chien, MD, Prof

Institute of Epidemiology and Preventive Medicine,College of Public Health,National Taiwan University, Taipei, Taiwan

Ana Rosa Cunha, PhD

State University of Rio de Janeiro, Rio de Janeiro, Brazil

Francois Dabis, MD, PhD, Prof.

Centre de Recherche INSERM U.897,Institut de Santé Publique, Epidémiologie et Développement (ISPAD),Université Victor Segalen Bordeaux 2, Bordeaux Cedex, France

Jesse Dawson, Dr.

Institute of Cardiovascular and Medical Sciences,University of Glasgow, Glasgow, UK

Ralph A. DeFronzo, MD

University of Texas Health Science Center, San Antonio, USA

Jaqueline .M. Dekker, Prof

Department of Epidemiology and Biostatistics,University Medical Center , Amsterdam, the Netherlands

and

EMGO Institute for Health and Care Research, VU University Medical Center, Amsterdam, the Netherlands

Moise Desvarieux, MD, PhD, Assoc. Prof.

Department of Epidemiology,Mailman School of Public Health,Columbia University, New York, USA

Chrystosomos Dimitriadis, MD

University Department of Nephrology, Hippokration General Hospital, Thessaloniki, Greece

Pierre Ducimetiere, Prof.

University Paris_Sud Xi, Kremlin-Bicêtre, Le Kremlin-Bicêtre, France

Marcus Dörr, MD, Prof.

Department B for Internal Medicine, University Medicine Greifswald, Greifswald, Germany

and

German Center for Cardiovascular Research (DZHK),partner site Greifswald , Greifswald, Germany

Helen Eddington, MD

University Hospitals Birmingham, Birmingham, UK

Robert Ekart, MD, PhD, Assist. Prof

Department of Dialysis,Clinic for Internal Medicine,University Medical Centre Maribor, Maribor, Slovenia

Jean Philippe Empana, MD, PhD

Paris Cardiovascular Research Centre (PARCC), University Paris Descartes, Sorbonne Paris Cité, UMR-S970, Paris, France

Gunnar Engström, MD, PhD, Prof.

Department of Clinical Sciences in Malmö, Lund University, Malmö, Sweden

Mark A. Espeland, PhD, Prof.

Department of Biostatistical Sciences, Wake Forest School of Medicine, Winston-Salem, NC, USA

Christine Espinola-Klein, MD, Prof.

2nd Department of Medicine, Johannes-Gutenberg University, Mainz, Germany

Ramon Estruch, MD, PhD

Endocrinology & Nutrition Service, Hospital Clínic, Barcelona, Spain

Thorleif Etgen, MD, PD

Department of Neurology,Kliniken Südostbayern,Klinikum Traunstein, Traunstein, Germany

and

Department of Psychiatry and Psychotherapy, Technische Universität München, Munich, Germany

Marat Ezhov, MD, PhD

Atherosclerosis Department,Cardiology Research Center , Moscow, Russia

Oscar H. Franco, MD, PhD, FESC, FFPH, Prof.

Department of Epidemiology, Erasmus MC, University Medical Center Rotterdam, Rotterdam, the Netherlands

Beat Frauchiger, MD, Prof.

Department of Internal Medicine,Division of Angiology,Kantonsspital Frauenfeld, Frauenfeld, Switzerland

Alfonsa Friera, MD

Radiology Department,Hospital Universitario de la Princesa,Universidad Autónoma de Madrid, Madrid, Spain

Rafael Gabriel, Prof., MD, Phd

Instituto de Investigación Princesa IP,Hospital Universitario de la Princesa,Universidad Autónoma de Madrid, Madrid, Spain

Greg Gamble, MSc

Department of Medicine, The University of Auckland, Auckland, New Zealand

Lu Gao, MSc

MRC Biostatistics Unit,Institute of Public Health,University Forvie Site, Cambridge, UK

Rachel Georgiou

Salford Royal Hospitals NHS Foundation Trust, Salford, UK

Hertzel C. Gerstein, MD, MSc

Department of Medicine and Population Health Research Institute,McMaster University , Hamilton, Ontario, Canada

Paolo Gresele, Prof, MD, PhD

Department of Internal,Division of Internal and Cardiovascular Medicine,University of Perugia, Perugia, Italy

Liliana Grigore, MD

Centro Sisa per lo Studio della Aterosclerosi, Bassini Hospital, Cinisello Balsamo, Italy

Diederick E. Grobbee, MD, PhD, Prof

Julius Center for Health Sciences and Primary Care, University Medical Center Utrecht, Utrecht, the Netherlands

Muriel P.C. Grooteman

Institute for Cardiovascular Research VU Medical Center (ICaR-VU),VU Medical Center, Amsterdam, The Netherlands

and

Department of Nephrology, VU Medical Center, Amsterdam, The Netherlands

Giuseppe Guglielmini, MD, PhD

Division of Internal and Cardiovascular Medicine,Department of Medicine,University of Perugia, Perugia, Italy

Félix Gutiérrez, MD, PhD

Clinical Medicine,Hospital General Universitario de Elche,University Miguel Hernández, Elche, Alicante, Spain

Markolf Hanefeld, Prof

Study Centre Professor Hanefeld, GWT,TU Dresden, Dresden, Germany

Apostolos I. Hatzitolios, MD, PhD, FESH, Prof.

Head of first1 Propedeutic Department of Internal Medicine,Medical School,Aristotle University of Thessaloniki - AHEPA Hospital, Thessaloniki, Greece

Bo Hedblad, MD, PhD, Prof

Department of Clinical Sciences in Malmö, Lund University, Malmö, Sweden

Frans A. Helmond, PhD

Merck Research Laboratories, Kenilworth, NJ, USA

Loghman Henareh, M.D., Ph.D., Prof.

Department of Medicine, Huddinge (MedH), H7,Karolinska Institutet , Stockholm, Sweden

Peter Higgins, Dr.

Institute of Cardiovascular and Medical Sciences,University of Glasgow, Glasgow, UK

Alan Hinderliter, MD

Department of Medicine, University of North Carolina, Chapel Hill, USA

Albert Hofman, MD, PhD, Prof.

Department of Epidemiology, Erasmus University Medical Center, Rotterdam, the Netherlands

Radovan Hojs, MD, PhD, Prof

Department of Nephrology,Clinic for Internal Medicine,University Medical Centre Maribor, Maribor, Slovenia

and

Faculty of Medicine, University of Maribor, Maribor, Slovenia

Hirokazu Honda, MD, PhD, Assoc. Prof.

Division of Nephrology,Department of Medicine,Showa University Koto Toyosu Hospital, Tokyo, Japan

Satoshi Hoshide, MD

Department of Medicine,Division of Cardiovascular Medicine,Jichi Medical University School of Medicine, Tochigi, Japan

Menno V. Huisman

Department of Thrombosis and Hemostasis, Leiden University Medical Center, Leiden, the Netherlands

Joseph Hung, Winthrop Professor, MBBS (hons) FRACP FACC

Department of Cardiovascular Medicine, Sir Charles Gairdner Hospital, Nedlands, Australia

and

School of Medicine and Pharmacology, University of Western Australia, Nedlands, Australia

Bernhard Iglseder, MD, Prof

Parcelsus Medical University, Salzburg, Austria

and

Department of Geriatric Medicine, Gemeinnützige Salzburger Landeskliniken Betriebsgesellschaft GmbH Christian-Doppler-Klinik, Salzburg, Austria

M. Arfan Ikram, MD, PhD, Assoc. Prof.

Department of Epidemiology, Erasmus University Medical Center, Rotterdam, the Netherlands

and

Department of Neurology, Erasmus University Medical Center, Rotterdam, the Netherlands

and

Department of Radiology, Erasmus University Medical Center, Rotterdam, the Netherlands

Raffaele Izzo, MD, Prof.

School of Medicine, Federico II University, Naples, Italy

Lisa M Jamieson, Assoc Prof

Australian Research Centre for Population Oral Health,School of Dentistry,The Univeristy of Adelaide, Adelaide, Australia

Tomas Jogestrand, M.D., Ph.D., Prof.

Department of Laboratory Medicine (LABMED), H5, Division of clinical physiology,Karolinska Universitetssjukhuset, Huddinge , Stockholm, Sweden

Stein Harald Johnsen, MD, PhD, Assoc. Prof.

Department of Clinical Medicine, University of Tromsø, Tromsø, Norway

and

Department of Neurology, University Hospital of Northern Norway, Tromsø, Norway

Aleksandar Jovanovic, MD, PhD, Prof

Faculty of Medicine, University of Prishtina, Prishtina\Kosovska Mitrovica, Serbia

Anna Kablak-Ziembicka, MD, PhD, Prof.

Department of Interventional Cardiology,Institute of Cardiology,Collegium Medicum Jagiellonian University, Krakow, Poland

Philip Kalra, MD, PhD, Prof.

Department of Renal Medicine,Manchester Academic Health Sciences Centre,Salford Royal Hospital, Manchester, UK

Kostas Kapellas

Australian Research Centre for Population Oral Health,School of Dentistry,The University of Adelaide, Adelaide, Australia

Kazuomi Kario

Department of Medicine,Division of Cardiovascular Medicine,Jichi Medical University School of Medicine, Tochigi, Japan

John JP Kastelein, MD, Prof

Department of Vascular Medicine,Academic Medical Center,University of Amsterdam, Amsterdam, the Netherlands

Akihiko Kato, MD, Prof.

Blood Purification Unit, Hamamatsu University Hospital, Hamamatsu, Japan

Jussi Kauhanen, MD, Prof

Institute of Public Health and Clinical Nutrition, University of Eastern Finland, Kuopio Campus, Kuopio, Finland

Maryam Kavousi, MD, PhD

Department of Epidemiology and Biostatistics, Erasmus Medical Center, Rotterdam, the Netherlands

Masanori Kawasaki, MD, PhD, Assoc. Prof

Department of Cardiology, Gifu University Graduate School of Medicine, Gifu, Japan

Kerstin Kempf, PhD

Düsseldorf Catholic Hospital Group (VKKD),West-German Centre of Diabetes and Health (WDGZ) , Düsseldorf, Germany

Stefan Kiechl, MD, Prof

Department of Neurology, Medical University Innsbruck, Innsbruck, Austria

Jang-Young Kim, MD PhD

Department of Cardiology,Institute of Genomic Cohort,College of Medicine Yonsei University, Wonju, Korea

Kazuo Kitagawa, MD, PhD

Department of Neurology, Tokyo Women's Medical University, Tokyo, Japan

Sverre E. Kjeldsen, MD, PhD, Prof.

Department of Cardiology, Ullevaal University Hospital, Oslo, Norway

Sang Back Koh, MD PhD

Preventive Medicine, College of Medicine,Institute of Genomic Cohort,Yonsei University, Wonju, Korea

Svetlana Kostic, MSc, MD

Primarius Institute for Therapy and Rehabilitation,"Niska Banja" , Nis, Serbia

Manuel F Landecho, MD, PhD

Department of Internal Medicine, University Clinic of Navarra, Navarra, Spain

Tatjana Lazarevic, MA

Faculty of Medicine, University of Kragujevac, Kragujevac, Serbia

Moo-Sik Lee, MD., PhD., Prof.

College of Medicine, Konyang University Hospital, Daejeon, Korea

and

Department of Preventive Medicine, Konyang University, Daejeon, Korea

Seung Hwan Lee, M.D., PhD, Prof.

Department of Cardiology,College of Medicine,Yonsei University, Wonju, Korea

Wattana Leowattana, Assoc. Prof.

Department of Clinical Tropical Medicine,Faculty of Tropical Medicine,Mahidol University, Rachatawee, Bangkok, Thailand

Ximing Liao, BSc, MSc, PhD

Department of Neurology, Goethe University, Frankfurt am Main, Germany

Hung-Ju Lin, MD

Department of Internal Medicine, National Taiwan University Hospital, Taipei, Taiwan

Yao-Ping Lin, MD

Taipei Veterans General Hospital, Taipei, Taiwan

Lars Lind, MD, PhD, Prof

Department of Medicine, Uppsala University, Uppsala, Sweden

Chun-Feng Liu, MD, PhD, Prof

Department of Neurology,Institute of Neuroscience,the Second Affiliated Affiliated Hospital of Soochow University, Soochow, China

Jing Liu, MD, PhD, Prof.

Department of Epidemiology,Beijing Institute of Heart, Lung and Blood Vessel Diseases,Beijing Anzhen Hospital, Capital Medical University, Beijing, China

Eva Lonn, MD, MSc, FRCPC, FACC, Prof.

Department of Medicine and Population Health Research Institute, McMaster University, Hamilton, Ontario, Canada

Matthias W. Lorenz, MD, Prof.

Department of Neurology, Goethe University, Frankfurt am Main, Germany

Dongmei Ma, MSc

Institute of Child and Adolescent Health, School of Public Health,Peking University, Beijing, China

Dianna Magliano, Assoc. Prof

BakerIDI Heart and Diabetes Institute , Melbourne, Australia

Stephan Martin, Prof. Dr.

Düsseldorf Catholic Hospital Group (VKKD),West-German Centre of Diabetes and Health (WDGZ) , Düsseldorf, Germany

Mar Masiá, MD, PhD

Cinical Medicine,Hospital General Universitario de Elche,University Miguel Hernández, Elche, Alicante, Spain

Ellisiv B. Mathiesen, MD, PhD, Prof.

Department of Clinical Medicine, University of Tromsø, Tromsø, Norway

and

Department of Neurology and Neurophysiology, University Hospital of Northern Norway, Tromsø, Norway

Wolfgang Mayer-Berger

Centre for Cardiovascular Rehabilitation, Leichlingen, Germany

Barry P. McGrath, Prof

Department of Vascular Sciences, Monash University, Dandenong Hospital, Melbourne, Australia

Stela McLachlan, PhD

Centre for Population Health Sciences,Usher Institute of Population Health Sciences and Informatics,University of Edinburgh, Edinburgh, UK

John McNeil, PhD, Prof., MBBS

School of Public Health and Preventive Medicine, Monash University, Melbourne, Australia

Brendan McQuillan, Assoc. Prof, MBBS PhD FRACP

Department of Cardiovascular Medicine, Sir Charles Gairdner Hospital, Nedlands, Australia

and

School of Medicine and Pharmacology, University of Western Australia, Nedlands, Australia

Patrick Mercié, Prof

Service de Médecine Interne,Hôpital Saint-André,CHU de Bordeaux, Bordeaux, France

Rino Migliacci, MD

Division of Internal Medicine,Ospedale della Valdichiana“S.Margherita” , Cortona, Italy

Dragan Mijalkovic, MD

Primarius,Polyclinic "Kardiomedika" , Nis, Serbia

Firouzeh Moeinzadeh, MD

Isfahan Kidney Disease Research Center,Isfahan University of Medical Sciences, Isfahan, Iran

Mojgan Mortazavi, MD, Assoc. Prof.

Nephrology departement,Isfahan Kidney Diseases Research Center,Isfahan University of medical sciences, Isfahan, Iran

Titus Francis Msoka, MD, PhD

Kilimanjaro Christian Medical Centre (KCMC) , Moshi, Tanzania

and

Kilimanjaro Clinical Research Institute (KCRI) , Moshi, Tanzania

Veronica A. Myasoedova, MD, PhD

Laboratory of Angiopathology,Institute of General Pathology and Pathophysiology , Moscow, Russia

Michiaki Nagai, MD

Department of Medicine,Division of Cardiovascular Medicine,Jichi Medical University School of Medicine, Tochigi, Japan

Tsukasa Nakamura, MD, PhD

Division of Nephrology,Department of Internal Medicine,Shinmatsudo Central General Hospital, Chiba, Japan

Prabath W.B. Nanayakkara

Department of Clinical Neurophysiology,Medical Center,VU University Amsterdam, Amsterdam, the Netherlands

Dariusz Naskret, MD, PhD

Department of Internal Medicine and Diabetology, Poznan University of Medical Sciences, Poznan, Poland

Mario Fritsch Neves, MD, PhD

State University of Rio de Janeiro, Rio de Janeiro, Brazil

Pythia T. Nieuwkerk, PhD

Department of Medical Psychology, Academic Medical Center, Amsterdam, the Netherlands

Giel Nijpels, MD, PhD

Department of General Practice,VU University Medical Center , Amsterdam, the Netherlands

and

EMGO Institute for Health and Care Research, VU University Medical Center, Amsterdam, the Netherlands

Giuseppe D. Norata, PhD

Dipartimento di Scienze Farmacologiche e Biomolecolari, Università degli Studi di Milano, Milan, Italy

and

SISA Center for the Study of Atherosclerosis, Bassini Hospital, Cinisello Balsamo, Italy

George Ntaios, MD, MSc (Stroke Med), PhD, FESO

Department of Medicine, University of Thessaly, Larissa, Greece

Shuhei Okazaki, MD

Department of Neurology, Osaka University Graduate School of Medicine, Osaka, Japan

Michael Hecht Olsen, MD, PhD, DMSc, Prof

Cardiovascular and Metabolic Preventive Clinic,Department of Endocrinology,Odense University Hospital, Odense, Denmark

Alexander N. Orekhov, PhD, DSc

Institute for atherosclerosis Research,Skolkovo Innovation Center, Moscow, Russia

and

Laboratory of Angiopathology,Institute of General Pathology and Pathophysiology , Moscow, Russia

Aikaterini Papagianni, MD, Assoc. Prof.

University Department of Nephrology, Hippokration General Hospital, Thessaloniki, Greece

Hyun Woong Park, M.D.

College of Medicine, Konyang University Hospital, Daejeon, Korea

Grace Parraga, Dr

Robarts Research Institute,Western University, London, Canada

Sharif Pasha

Department of General Internal Medicine, Leiden University Medical Center, Leiden, the Netherlands

Matthieu Plichart, MD, PhD

Assistance Publique, Hôpitaux de Paris, Hôpital Broca, Paris, France

and

Paris Cardiovascular Research Centre (PARCC), University Paris Descartes, Sorbonne Paris Cité, UMR-S970, Paris, France

Janice Pogue, PhD

Population Health Research Institute, McMaster University, Hamilton, Ontario, Canada

Joseph F. Polak, MD, MPH, Prof

Tufts University School of Medicine, Tufts Medical Center, Boston, USA

Holger Poppert, MD, PhD

Department of Neurology, Technische Universität München, Munich, Germany

David Preiss, MD, PhD

BHF Glasgow Cardiovascular Research Centre, University of Glasgow, Glasgow, UK

Jackie F. Price, MD

Centre for Population Health Sciences,Usher Institute of Population Health Sciences and Informatics,University of Edinburgh, Edinburgh, UK

Tadeusz Przewlocki, MD, PhD, Prof.

Department of Interventional Cardiology,Institute of Cardiology,Collegium Medicum Jagiellonian University, Krakow, Poland

Joel Raichlen, MD

AstraZeneca, Wilmongton, DE, USA

Peter Reaven, MD, Prof.

Dept. of Medicine,Phoenix VA Health Care System,University of Arizona, Phoenix, USA

Peter Reiss, MD, PhD, Prof.

Amsterdam Institute for Global Health and Development, University of Amsterdam, Amsterdam, the Netherlands

and

Department of Global Health, Academic Medical Center, Amsterdam, the Netherlands

Christine Robertson, MBChB

Centre for Population Health Sciences,Usher Institute of Population Health Sciences and Informatics,University of Edinburgh, Edinburgh, UK

Kimmo Ronkainen, MSc

Institute of Public Health and clinical Nutrition, University of Eastern Finland, Kuopio Campus, Kuopio, Finland

Emilio Ros, MD, PhD

Endocrinology & Nutrition Service, Hospital Clínic, Barcelona, Spain

Signe Rosenlund, MD

Steno Diabetes Center , Gentofte, Denmark

Peter Rossing, MD, DMSc, Prof.

Steno Diabetes Center , Gentofte, Denmark

Maria Rosvall, MD, PhD, Assoc. Prof.

Department of Clinicla Sciences in Malmö, Lund University, Malmö, Sweden

Francesco Rozza, MD, Prof.

Department of Medicine and Surgery, University of Salerno, Salerno, Italy

Tatjana Rundek, MD, PhD, Prof.

Department of Neurology,Miller School of Medicine,University of Miami, Miami, USA

Mohammad Saadatnia, MD, Assoc. Prof.

Al-Zahra Hospital, Isfahan University of Medical Sciences, Isfahan, Iran

Ralph L. Sacco, MD, MS, Prof.

Department of Neurology,Miller School of Medicine,University of Miami, Miami, USA

Maya Safarova, M.D.

Atherosclerosis Department,Cardiology Research Center , Moscow, Russia

Dirk Sander, MD, Prof

Department of Neurology, Benedictus Hospital Tutzing & Feldafing, Feldafing, Germany

and

Department of Neurology, Technische Universität München, Munich, Germany

Kerstin Sander, MD, PD

Department of Psychosomatic,Schön Klinik , Berchtesgadener Land, Germany

Eiichi Sato, MD

Division of Nephrology,Department of Internal Medicine , Chiba, Japan

Naveed Sattar, MD, PhD, Prof

BHF Glasgow Cardiovascular Research Centre, University of Glasgow, Glasgow, UK

Christos Savopoulos, MD, PhD, Assoc. Prof

1st Propedeutic Department of Internal Medicine, Aristotles University of Thessaloniki, Thessaloniki, Greece

Frank Scheckenbach, MSc, PhD

Department of Neurology, Goethe University, Frankfurt am Main, Germany

Caroline Schmidt, PhD, Assoc. Prof.

Walleng Laboratory for Cardiovascular Research, University of Gothenburg, Gothenburg, Sweden

Irene Schmidtmann, Dr.

Institut fuer Medizinische Biometrie, Epidemiologie und Informatik (IMBEI), Universitaetsmedizin Mainz, Mainz, Germany

Ulf Schminke, MD, Prof

Department of Neurology, Greifswald University Clinic, Greifswald, Germany

Michael Schneider, Dr.

Dept HR Services & Expertise Center,Boehringer Ingelheim Pharma GmbH & Co. KG , Ingelheim am Rhein, Germany

and

Mannheim Institute for Public Health,Medical Faculty Mannheim,Ruprecht-Karls University Heidelberg, Mannheim, Germany

Ercan Sevinc Ok, MD

Division of Nephrology, Izmir Bozyaka Education and Research Hospital, Izmir, Turkey

Norman Sharpe, MD, Prof.

Heart Foundation, Ellerslie, Auckland, New Zealand

Patrick Sheridan, MSc

Population Health Research Institute, McMaster University, Hamilton, Ontario, Canada

Andrew Sherwood, PhD

Department of Psychiatry and Behavioral Sciences, Duke University Medical Center, Durham, NC 27710, USA

Cesare R. Sirtori, MD, PhD, Prof.

Center of Dyslipidemias,Niguarda Ca’ Granda Hospital, Milano, Italy

Matthias Sitzer, MD, Prof.

Department of Neuology, Klinikum Herford, Herford, Germany

and

Department of Neurology, Goethe University, Frankfurt am Main, Germany

Michael Skilton, PhD

Boden Institute of Obesity, Nutrition, Exercise and Eating Disorders, University of Sydney, Sydney, Australia

Patrick J Smith, PhD, MPH

Department of Psychiatry and Behavioral Sciences, Duke University Medical Center, Durham, NC 27710, USA

Igor A. Sobenin, MD, PhD, DSc

Laboratory of Angiopathology,Institute of General Pathology and Pathophysiology , Moscow, Russia

and

Laboratory of Medical Genetics, Department of Cardiovascular Pathology,AM Myasnikov Institute of Clinical Cardiology,Russian Cardiology Research and Production Complex, Moscow, Russia

J. David Spence, MD, FRCPC, FAHA

Stroke Prevention & Atherosclerosis Research Centre,Robarts Research Institute,Western University, London, Canada

Sathanur R. Srinivasan, PhD, Prof.

Center for Cardiovascular Health, Department of Epidemiology, Biochemistry, Tulane University School of Public Health and Tropical Medicine, New Orleans, USA

Daniel Staub, MD, Prof.

Department of Angiology, University Hospital Basel, Basel, Switzerland

CDA Stehouwer, MD, PhD, FESC

Department of Internal Medicine and Cardiovascular Research Institute Maastricht (CARIM), Maastricht University Medical Centre, Maastricht, the Netherlands

Helmuth Steinmetz, MD, Prof

Department of Neurology, Goethe University, Frankfurt am Main, Germany

Radojica Stolic, MD, PhD, Prof

Faculty of Medicine, University of Kragujevac, Kragujevac, Serbia

Erik Stroes, MD, PhD

Department of Vascular Medicine, Academic Medical Center, Amsterdam, the Netherlands

Ta-Chen Su, MD, PhD, Assoc. Prof.

Department of Internal Medicine, National Taiwan University Hospital, Taipei, Taiwan

Carmen Suarez, MD, PhD

Internal Medicine Department,Hospital Universitario de la Princesa,Universidad Autónoma de Madrid, Madrid, Spain

Ivan S. Tasic, MD, PhD, Prof.

Faculty of Medicine,Institute for Therapy and Rehabilitation,University of Nis, "Niska Banja", Serbia

Rodolphe Thiébaut, Dr.

Centre de Recherche INSERM U.897,Institut de Santé Publique, Epidémiologie et Développement (ISPAD),Université Victor Segalen Bordeaux 2, Bordeaux Cedex, France

Peter L. Thompson, Clinical Professor, MD, FRACP, FACC, MBA

Heart Research Institute of WA and Department of Cardiovascular Medicine, Sir Charles Gairdner Hospital, Nedlands, Australia

Simon G. Thompson, DSc, Prof.

Department of Public Health and Primary Care,School of Clinical Medicine,University of Cambridge, Cambridge, UK

Estefania Toledo, MD, MPH, PhD

Centro de Investigación Biomédica en Red-Fisiopatología de la Obesidad y la Nutrición (CIBERobn), , Spain

and

Department of Preventive Medicine and Public Health, University of Navarra, Pamplona, Spain

Elena Tremoli, PhD, Prof

Centro Cardiologico Monzino, IRCCS, Milan, Italy

and

Dipartimento di Scienze Farmacologiche e Biomolecolari, Università di Milano, Milan, Italy

Devjit Tripathy, MD, PhD

University of Texas Health Science Center, San Antonio, USA

Giovanni Tripepi, MSc

Clinical Epidemiology and Pathophysiology of Renal Diseases and Hypertension Unit, Institute of Physiology of the National Research Council (CNR), Reggio Calabria, Italy

Tomi-Pekka Tuomainen, MD, PhD, Prof

Institute of Public Health and Clinical Nutrition, University of Eastern Finland, Kuopio Campus, Kuopio, Finland

Aleksandra Uruska, MD, PhD

Department of Internal Medicine and Diabetology, Poznan University of Medical Sciences, Poznan, Poland

Heiko Uthoff, MD

Department of Angiology, University Hospital Basel, Basel, Switzerland

Fabrizio Veglia, PhD

Centro Cardiologico Monzino, IRCCS, Milan, Italy

Frank L.J. Visseren, MD, Prof

Department of Vascular Medicine, University Medical Centre Utrecht, Utrecht, the Netherlands

Henry Völzke, MD, Prof

German Center for Cardiovascular Research (DZHK),partner site Greifswald , Greifswald, Germany

and

Institute for Community Medicine, SHIP/Clinical-Epidemiological Research, Greifswald, Germany

Kristian Wachtell, MD, PhD, Assoc. Prof.

Department of Cardiology, Gentofte University Hospital, Copenhagen, Denmark

Matthew Walters, Prof.

Institute of Cardiovascular and Medical Sciences,University of Glasgow, Glasgow, UK

Zhenghe Wang, MD

Institute of Child and Adolescent Health, School of Public Health,Peking University, Beijing, China

Thapat Wannarong, Dr

Stroke Prevention & Atherosclerosis Research Centre,Robarts Research Institute,Western University, Mahidol University, London/Bangkok, Canada/Thailand

Gillian Whalley, PhD, Prof.

Faculty of Social and Health Sciences, Unitec, Auckland, New Zealand

Johann Willeit, MD, Prof.

Department of Neurology, Medical University Innsbruck, Innsbruck, Austria

Peter Willeit, PhD

Department of Neurology, Medical University Innsbruck, Innsbruck, Austria

and

Department of Public Health and Primary Care,School of Clinical Medicine,University of Cambridge, Cambridge, UK

Miles D. Witham, Dr.

Ninewells Hospital,Ageing and Health Ninewells Hospital,Ninewells Hospital & Medical School, Dundee, UK

Wuxiang Xie, MD, PhD, Assist. Prof.

Department of Epidemiology,Beijing Institute of Heart, Lung and Blood Vessel Diseases,Beijing Anzhen Hospital, Capital Medical University, Beijing, China

Kiyofumi Yamada, MD, PhD

Departments of Neurosurgery, Gifu University Graduate School of Medicine, Gifu, Japan

David N. Yanez, PhD, Assoc. Prof

Department of Biostatistics, University of Washington, Seattle, USA

Shinichi Yoshimura, MD, PhD, Assoc. Prof.

Departments of Neurosurgery, Gifu University Graduate School of Medicine, Gifu, Japan

Wen-Chung Yu, MD; Assoc. Prof

National Yang-Ming University, Taipei, Taiwan

and

Division of Cardiology, Taipei Veterans General Hospital, Taipei, Taiwan

Salim Yusuf, MD, Dphil

Department of Medicine and Population Health Research Institute, McMaster University, Hamilton, Ontario, Canada

Dong Zhao, MD, PhD, Prof.

Department of Epidemiology,Beijing Institute of Heart, Lung and Blood Vessel Diseases,Beijing Anzhen Hospital, Capital Medical University, Beijing, China

Carmine Zoccali, MD, Prof.

Clinical Epidemiology and Pathophysiology of Renal Diseases and Hypertension Unit, Institute of Physiology of the National Research Council (CNR), Reggio Calabria, Italy

Zhi-Yong Zou

Institute of Child and Adolescent Health, School of Public Health,Peking University, Beijing, China

Sophia Zoungas, Assoc. Prof.

School of Public Health and Preventive Medicine, Monash University, Melbourne, Australia

Dorota A. Zozulinska-Ziólkiewicz, MD, PhD, Prof.

Department of Internal Medicine and Diabetology, Poznan University of Medical Sciences, Poznan, Poland

Eric de Groot, MD, PhD

Imagelabonline & Cardiovascular,Eindhoven and Clinical Epidemiology and Biostatistics,Academic Medical Centre, Amsterdam, the Netherlands

Nicola de Luca, MD, Prof.

School of Medicine, Federico II University, Naples, Italy

Pieter M. ter Wee, Prof Dr.

Department of Nephrology,Medical Center,VU University Amsterdam, Amsterdam, the Netherlands

Michiel A. van Agtmael, MD, PhD

Department of Internal Medicine, VU University Medical Center, Amsterdam, the Netherlands

Wiek van Gilst, PhD, Prof

Department of Experimental Cardiology, University Medical Center Groningen, Groningen, the Netherlands

Marit G. A. van Vonderen, MD, PhD

Medical Center Leeuwarden, Leeuwarden, the Netherlands

Statistical Advisor:

Simon Thompson, DSc, Prof

Department of Public Health and Primary Care, School of Clinical Medicine,University of Cambridge, Cambridge, UK

Principal Investigator:

Matthias W. Lorenz, MD, Prof.

Department of Neurology, Goethe University, Frankfurt am Main, Germany
